# Mutation-based mechanism and evolution of the potent multidrug efflux pump RE-CmeABC in *Campylobacter*

**DOI:** 10.1073/pnas.2415823121

**Published:** 2024-11-27

**Authors:** Lei Dai, Zuowei Wu, Orhan Sahin, Shaohua Zhao, Edward W. Yu, Qijing Zhang

**Affiliations:** ^a^Department of Veterinary Microbiology and Preventive Medicine, College of Veterinary Medicine, Iowa State University, Ames, IA 50011; ^b^Department of Veterinary Diagnostic and Production Animal Medicine, College of Veterinary Medicine, Iowa State University, Ames, IA 50011; ^c^Center for Veterinary Medicine, U.S. Food and Drug Administration, Laurel, MD 20708; ^d^Department of Pharmacology, School of Medicine, Case Western Reserve University, Cleveland, OH 44106

**Keywords:** multidrug efflux, Campylobacter, antibiotic resistance, pathogen evolution, gene editing

## Abstract

The resistance-nodulation-cell division (RND) superfamily of multidrug efflux systems are important players in mediating antibiotic resistance in gram-negative pathogens. *Campylobacter jejuni*, a major enteric pathogen, utilizes an RND-type transporter system, CmeABC, as the primary mechanism for extrusion of various antibiotics. Recently, a functionally potent variant of CmeABC (named RE-CmeABC) emerged in clinical *Campylobacter* isolates, conferring enhanced resistance to multiple antibiotic classes. Despite the clinical importance of RE-CmeABC, the molecular mechanisms for its functional gain and its evolutionary trajectory remain unknown. Here, we demonstrated that amino acid substitutions in RE-CmeB (inner membrane transporter), but not in RE-CmeA (periplasmic protein) and RE-CmeC (outer membrane protein), in conjunction with a nucleotide mutation in the promoter region of the efflux operon, are responsible for the functional gain of the multidrug efflux system. We also showed that RE-*cmeABC* is emerging globally and distributed in genetically diverse *C. jejuni* strains, suggesting its possible spread by horizontal gene transfer. Notably, many of RE-*cmeABC* harboring isolates were associated with the human host including strains from large disease outbreaks, indicating the clinical relevance and significance of RE-CmeABC. Evolutionary analysis indicated that RE-*cmeB* likely originated from *Campylobacter coli*, but its expansion mainly occurred in *C. jejuni,* possibly driven by antibiotic selection pressure. Additionally, RE-*cmeB*, but not RE-*cmeA* and RE-*cmeC*, experienced a selective sweep and was progressing to be fixed during evolution. Together, these results identify a mutation-based mechanism for functional gain in RE-CmeABC and reveal the key role of RE-CmeB in facilitating *Campylobacter* adaptation to antibiotic selection.

## Significance

A major mechanism utilized by *Campylobacter* for antibiotic resistance is the CmeABC efflux system that reduces antibiotic accumulation in bacterial cells and hence compromises the efficacy of antibiotic therapy. This study reveals how *Campylobacte*r alters the sequences of CmeABC to enhance its function in antibiotic resistance and how this potent efflux system is evolved in *Campylobacter* in response to antibiotic selection pressure. The findings define a mechanism underlying *Campylobacter* adaptation to antibiotic selection and may facilitate the development of strategies to control antibiotic-resistant *Campylobacter*. Additionally, the gene replacement method developed in this study may be adapted for understanding the functions of genes of interest in *Campylobacter* and other bacterial species.

The rapid emergence and continued spread of antibiotic-resistant bacteria have compromised the efficacy of antibiotics in treating infectious diseases, which has become a public health crisis worldwide (1–4). Bacterial pathogens utilize various means for mediating antibiotic resistance. Among the known mechanisms, multidrug efflux pumps are important players and confer resistance to structurally diverse classes of antibiotics by reducing intracellular antibiotic accumulation in bacterial cells (5, 6). In gram-negative bacteria, members of the resistance-nodulation-cell division (RND) superfamily are the most clinically relevant in mediating resistance to antibiotics (6, 7). These RND-type transporters are tripartite systems spanning both the inner and outer membranes, including an inner membrane transport protein, an outer membrane channel protein, and a periplasmic fusion protein (6). These RND transporters contribute to both intrinsic and acquired resistance to antibiotics and are often regulated by transcriptional repressors (7, 8). Overexpression of the transporter systems, mediated by alteration of promoter activities, and functional gain resulting from point mutations in the inner membrane transporter contribute to bacterial acquired resistance to antibiotics (7, 9, 10).

*Campylobacter jejuni* is a leading cause of foodborne bacterial gastroenteritis worldwide (11) and is responsible for an estimated 1.3 million cases of illnesses each year in the United States (12). Antibiotic therapy is necessary for severe cases of enteritis or in immunocompromised patients (13); however, *Campylobacter* is increasingly resistant to clinically important antimicrobials, such as fluoroquinolones, and new antibiotic resistance mechanisms continue to emerge in this organism (14–17). As an RND-type efflux pump, CmeABC is the primary antibiotic extrusion system in *Campylobacter* and plays a critical role in resistance to a broad range of antimicrobials and toxic compounds (18, 19). This tripartite efflux pump is encoded by the *cmeABC* operon and includes the CmeB inner membrane transport protein, the CmeA periplasmic fusion protein, and the CmeC outer membrane protein, which function together to form an antibiotic extrusion apparatus across the *Campylobacter* cell membrane. In addition to mediating antibiotic resistance, CmeABC has an important natural function in bile resistance and is essential for *C. jejuni* colonization in the intestinal tract (18). The fact that CmeABC is ubiquitously distributed in *C. jejuni* and *C. coli* strains further indicates its essential role in facilitating *Campylobacter* adaptation in animal hosts. The *cmeABC* operon is regulated by a transcriptional regulator named CmeR, which binds specifically to an inverted repeat (IR) in the promoter region of the operon and inhibits the expression of *cmeABC* (20). Mutations in the IR or deletion of *cmeR* result in overexpression of *cmeABC*, which only produces a modest increase in the minimal inhibitory concentrations (MICs) of antibiotics (20).

Recently, a functionally enhanced variant (Resistance Enhancing CmeABC: RE-CmeABC) of CmeABC emerged in *Campylobacter* (21). RE-CmeABC confers significantly enhanced resistance to multiple antibiotics and shifts antibiotic MIC distributions to a much higher range among clinical isolates (21), indicating its key influence on *Campylobacter* susceptibility to antibiotics. For example, in the presence of RE-CmeABC and a GyrA mutation, *Campylobacter* expresses an exceedingly high resistance level to ciprofloxacin (MIC ≥ 256 µg/ml) (21), which is ≥16-fold higher than the GyrA mutant strains harboring a typical CmeABC. Based on amino acid sequence alignments, RE-CmeABC is divergent from the typical CmeABC homologs in both *C. jejuni* and *C. coli* (21). Thus, RE-CmeABC represents a potent antibiotic efflux mechanism in *Campylobacter*. The clinical significance of RE-CmeABC was further corroborated by several recent studies (22–25), which demonstrated the increasing prevalence of RE-*cmeABC* in isolates derived from both food-producing animals and human patients including disease outbreak strains in different geographical regions of the world.

Despite the importance of RE-CmeABC in antibiotic resistance, the molecular and evolutionary mechanisms for its emergence and enhanced function remain unknown. Compared to the typical wild-type *cmeABC* operon in *C. jejuni* NCTC11168, RE-*cmeABC* harbors 595 single nucleotide polymorphisms (SNPs) in RE-*cmeB*, 201 SNPs in RE-*cmeC*, and 16 SNPs in RE-*cmeA,* which results in a number of amino acid changes in the encoded products (21). However, it is unclear whether the mutations in all three genes contribute to its enhanced function in antibiotic resistance. Phylogenetically, RE-*cmeB* sequences formed a unique subtree, which is apart from the other *cmeB* sequences of *C. jejuni* and *C. coli* (21), but how RE-*cmeB* was evolved remains unknown. To address these gaps and generate information that may facilitate the control of antibiotic-resistant *Campylobacter*, we utilized a high-throughput screening strategy and CRISPR-Cas9 (CRISPR, CRISPR-Cas and CRISPR-associated protein 9) based gene replacement to identify the mutations in RE-*cmeB* and the promoter of RE-*cmeABC* required for the enhanced function of RE-CmeABC in multidrug resistance. Subsequently, we analyzed the distribution and evolution of RE-*cmeB* in *Campylobacter* isolates by using the *Campylobacter* genome sequences deposited in the NCBI Pathogen Detection Database. Our results revealed that amino acid substitutions in RE-CmeB, but not in CmeA and CmeC, played a major role in mediating the enhanced function of RE-CmeABC, although a mutation in the promoter also contributed to the overall level of resistance. Our findings also suggest that RE-*cmeB* originated from *C. coli* despite its predominant expansion in *C. jejuni*. Additionally, evolutionary analysis indicated RE-*cmeB*, but not RE-*cmeA* and RE-*cmeC*, experienced selective sweeps and was fixed during evolution, further underlying the key role of RE-*cmeB* mutations in *Campylobacter* adaptation to antibiotic selection pressure.

## Results

### Transformation-Based Localization of the Regions Associated with the Enhanced Function of RE-CmeABC.

**Fig. 1.**
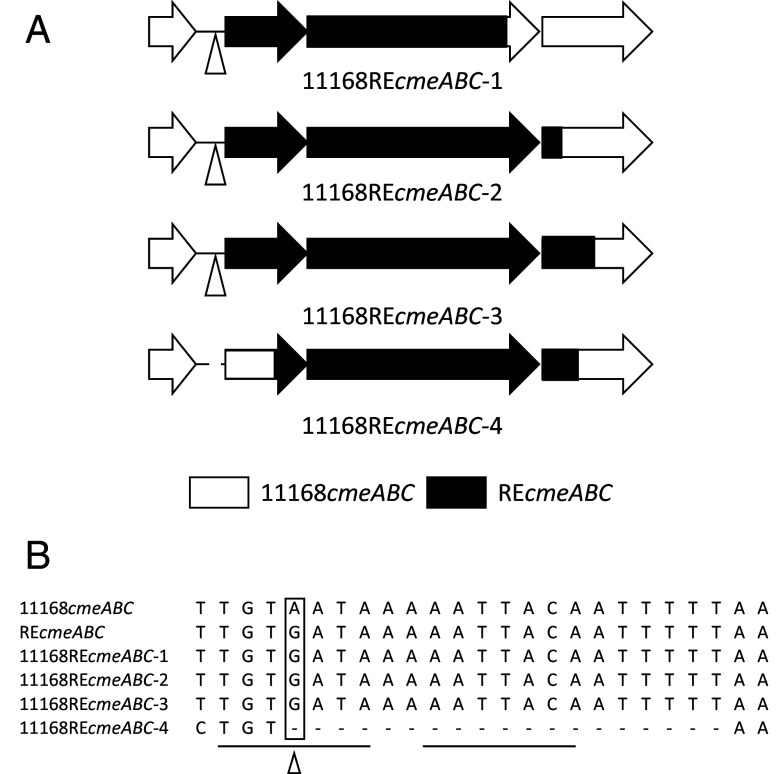
Transfer of RE-*cmeABC* into *C. jejuni* NCTC11168 by natural transformation. (*A*) Diagrams of the *cmeABC* region in different *C. jejuni* transformants. Open box arrows represent *cmeABC* sequences in wild-type NCTC11168, while solid box arrows represent RE-*cmeABC* sequences. The triangle in the promoter region represents the A to G point mutation. The gap before the *cmeA* gene of 11168RE*cme*ABC-4 represents a truncation in the *cmeA* promoter region. (*B*) Illustration of the CmeR binding site in the *cmeABC* promoter region. The location of the A–G mutation is indicated by a triangle. The inverted repeats serving as the CmeR binding site are underlined. The dashed line depicts deletions in the promoter region of 11168RE*cme*ABC-4.

To begin to map the RE-*cmeABC* sequences essential for the enhanced antibiotic resistance function, a PCR product containing the entire RE-*cmeABC* operon was amplified from *C. coli* DH161 (21), and its flanking sequences (including the promoter region) were electroporated into *C. jejuni* NCTC11168 (Table 1), which harbors a typical *cmeABC* and has a florfenicol MIC of 0.5 µg/ml. Transformants that had acquired RE-*cmeABC* were selected by using 4 µg/ml florfenicol as RE-CmeABC confers enhanced resistance to this antibiotic (21). Four randomly picked transformants (11168RE*cme*ABC1 to 4; Table 1 and Fig. 1) were analyzed by antimicrobial susceptibility testing (AST), which showed 4- to 16-fold increases in the MICs of the tested antibiotics, including fluoroquinolones, macrolides, clindamycin, florfenicol, and tetracycline, except gentamicin (Table 2). DNA sequencing analysis of the *cmeABC* locus of the four transformants revealed that not all the point mutations in RE-*cmeABC* were transferred to the resistance-enhanced transformants (Fig. 1*A*). Specifically, transformants 1 to 3 gained most of the SNPs from RE-*cmeA* and RE-*cmeB,* but only part of, or no SNPs from RE-*cmeC*. In contrast, transformant 4 gained all SNPs from RE-*cmeB*, but only part of the SNPs from RE-*cmeA* and RE-*cmeC* (Fig. 1*A*). Additionally, an A–G mutation in the IR region of the *cmeABC* promoter was also transferred into transformants 1 to 3, and a truncation occurred in the IR in transformant 4 (Fig. 1*B*), resulting in an imperfect IR or disappearance of the IR of the CmeR-binding site (20). Of note, all four selected transformants had the same level of changes in the MICs of tested antibiotics (Table 2). It has been known that disruption of the CmeR-binding site releases its inhibition on the *cmeABC* promoter and results in overexpression of this efflux operon in *Campylobacter* (20). Together, the results suggested that mutations in the CmeR-binding site and the SNPs in RE-*cmeB* are necessary for the enhanced function of RE-CmeABC.

**Table 1.** *C. jejuni* strains and plasmid constructs used in this study

Strain or plasmid	Relevant genotype or phenotype	source
NCTC11168	*C. jejuni* wild-type isolate	(26)
11168RE*cmeABC*-1 to 4	NCTC11168 transformants from electroporation using RE-*cmeABC* as the DNA donor	This study
11168∆*cmeR*	NCTC11168 derivative with a Δ*cmeR*::*cat* insertional mutation	(20)
11168_A–G_	NCTC11168 derivative with the A–G mutation in the *cmeA* promoter sequence	This study
11168RE*cmeB*	NCTC11168 derivative with the *cmeB* replaced by the RE-*cmeB* sequences and a *cas9* and kanamycin resistance cassette *aph* inserted in the rRNA region	This study
11168_A–G_RE*cmeB*	11168_A–G_ derivative with the *cmeB* replaced by the RE*cmeB* sequences and a *cas9* and *aph* cassette inserted in the rRNA region	This study
11168_control_	NCTC11168 derivative with a *cas9* and *aph* cassette sequences inserted in the rRNA region	This study
pUC18	*E. coli* cloning vector containing an ampicillin resistance cassette *ampR*	(27)
pRRK	*C. jejuni* suicide plasmid for making gene insertions in the rRNA region	(28)
pRRK-cjCRISPR	A 10,485 bp plasmid originating from pRRK containing *cas9*, *cas1*, *cas2*, tracrRNA, and one crRNA repeat sequence from NCTC11168	This study
pCTarget-cas9	A 9,178 bp plasmid originating from pRRK-cjCRISPR, with the *cas1* and *cas2* sequences removed	This study
pCTarget-cas9-*cmeB*	The targeting plasmid originating from pCTarget-cas9 containing a 30 bp spacer sequence from *cmeB* of NCTC11168	This study
pRE*cmeB*	The editing plasmid originating from pUC18 containing the RE-*cmeB* replacement sequences	This study
pCTarget-*cmeB*	The targeting plasmid originating from pCTarget containing a 30 bp spacer sequence from *cmeB* of NCTC11168	This study
pcmeAP	A plasmid originating from pUC18 containing the flanking sequences of the CmeR-binding site in the *cmeA* promoter region	This study
pcmeAP_A–G_	A *C. jejuni* suicide plasmid originating from pcmeAP containing the A–G mutation in the *cmeA* promoter region	This study

**Table 2.** Antimicrobial MICs (µg/ml) of *C. jejuni* NCTC11168 and its RE-*cmeABC* transformants

Antibiotics	NCTC11168	11168RE*cmeABC*-1 to -4^*^	Fold increase of MIC
Azithromycin	0.03	0.12	4
Clindamycin	0.06	0.25	4
Ciprofloxacin	0.06	0.5	8
Erythromycin	0.25	1	4
Florfenicol	0.5	8	16
Gentamicin	0.5	0.5	–
Nalidixic acid	≤4	16	≥4
Telithromycin	0.5	2	4
Tetracycline	0.12	1	8

^*^The four transformants 11168RE*cmeABC*-1 to -4 exhibited identical MIC values to the tested antibiotics. “–” indicated no change in MIC.

### High-Throughput Mapping Identifies SNPs in RE-*cmeB* Essential for Enhanced Resistance.

**Fig. 2.**
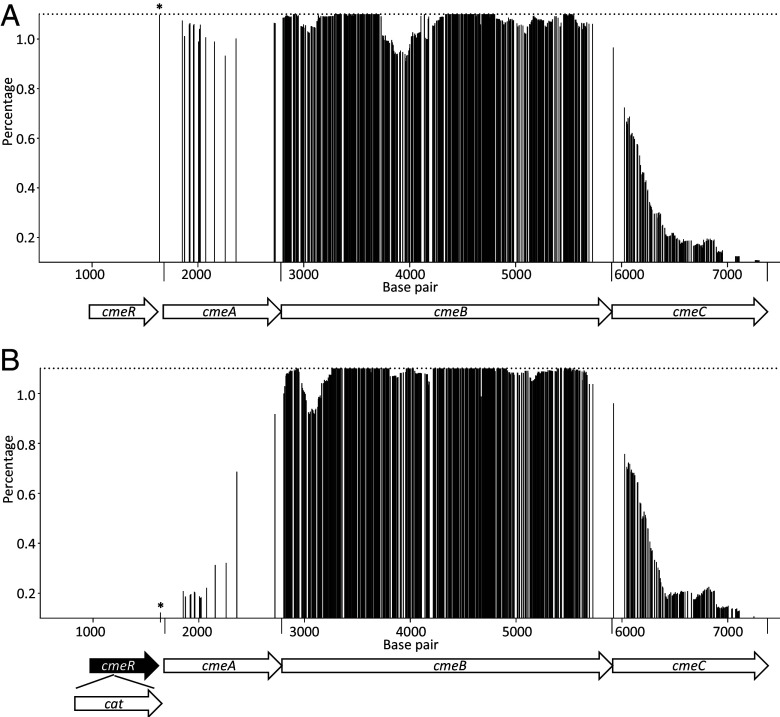
Frequencies of RE-*cmeABC* specific SNPs transferred into the *cmeABC* locus of the gain-of-function transformants in wild-type *C. jejuni* NCTC11168 (*A*) and its *cmeR*-deleted mutant (11168Δ*cmeR*) (*B*). The Y axis represents the frequencies of the RE-*cmeABC* alleles transferred at a given location. The X axis represents the coding sequences for *cmeABC* and its flanking regions. ORFs are depicted at the bottom of the panel. In both *A* and *B*, the asterisk indicates the position of the A–G mutation in the *cmeA* promoter region.

Given that there were numerous amino acid changes in RE-CmeABC compared with the typical CmeABC, it was not feasible to analyze their contributions to the enhanced function individually. Thus, we developed a high-throughput method to identify their roles by determining the transferred SNPs in the transformants that gained function in antibiotic resistance. The underlying hypothesis was that the SNPs that are required for the functional gain must be present in all of the transformants that appeared on antibiotic-containing plates. For this purpose, 273 individual transformants (individual colonies) that were selected on MH plates containing 4 µg/ml florfenicol were pooled and their genomic DNA was purified and subjected to whole genome sequence (WGS) analysis by the Illumina MiSeq system. The sequence reads were mapped against the *C. jejuni* NCTC11168 genome sequence. The results revealed that over 800 SNPs were transferred to the *cmeABC* region with varied frequencies (Fig. 2*A*). In total, 359 point mutations within several regions of RE-*cmeB* were uniformly (>99.5% transfer frequency considering the sequencing errors) detected in the transformants, suggesting their essential role in the enhanced function. These essential point mutations correspond to 99 amino acid changes (SI Appendix, Fig. S1 and Dataset S1). Although there were a large numbers of point mutations present in the RE-*cmeC* sequence, they were transferred at much lower frequencies (0 to 88.6%) than those in the RE-*cmeB* sequence, and no SNPs in RE-*cmeC* were transferred at >99.5% frequencies (Fig. 2*A*). Interestingly, the point mutation (A to G) in the promoter region of *cmeA* showed a 99.1% transfer frequency (Fig. 2*A*), while the point mutations within the coding sequence of RE-*cmeA* were transferred at frequencies 88.1 to 98.8%, suggesting that the promoter mutation, not the SNPs in RE-*cmeA*, is necessary for the enhanced resistance.

Since mutations in the IR were known to inhibit CmeR binding and resulted in overexpression of *cmeABC* (20, 29, 30), we utilized an 11168∆*cmeR* mutant strain as the recipient strain for transformation. In 11168∆*cmeR*, the repressor regulator CmeR was inactivated and *cmeABC* was overexpressed, making the A–G mutation at the CmeR binding site irrelevant for the expression of *cmeABC* (20). The RE-*cmeABC* PCR products were used as donor DNA to transform 11168∆*cmeR* and transformants were selected on florfenicol-containing plates. WGS was then used to analyze a pool of resistance-enhanced transformants (550 individual colonies). As shown in Fig. 2*B*, point mutations in RE-*cmeB* remained transformed at much higher frequencies (85.1 to 100%) than the mutations in RE-*cmeA* and RE-*cmeC*. Notably, a total of 399 point mutations within several regions of RE-*cmeB* were uniformly (>99.5% transfer frequency) detected in the transformants. These point mutations correspond to 112 amino acid changes (SI Appendix, Fig. S1 and Dataset S1). However, the transfer frequencies of the A–G mutation in the *cmeA* promoter and the point mutations in RE-*cmeA* were significantly reduced (3.2% for the A–G mutation and 15.8 to 90.1% for other mutations in RE-*cmeA*) compared to the transfer frequencies in wild-type NCTC11168 (Fig. 2*A*), indicating that the A–G mutation in the IR was no longer needed for the enhanced function of CmeABC in the 11168ΔcmeR background. The result also suggests that the mutations within the coding sequence of RE-*cmeA* and RE-*cmeC* were not essential for the enhanced function. Comparison of the results from the two separate transformation experiments revealed that 280 SNPs within RE-*cmeB* were uniformly (>99.5% transfer frequency) detected in both NCTC11168 and 11168ΔcmeR transformants, which corresponded to 75 amino acid changes (SI Appendix, Fig. S1 and Dataset S1), indicating their key role in the enhanced function. Detailed transfer frequencies of SNPs of RE-*cmeB* into the pooled transformants are listed in Dataset S2. Together, these results strongly suggest that sequence variations in RE-*cmeB* (not those in RE-*cmeA* and RE-*cmeC*) along with the A–G mutation in the CmeR binding site are most likely responsible for the enhanced efflux function of RE-CmeABC.

### Gene Replacement Mediated by the CRISPR-Cas9 System Confirms the key Role of RE-*cmeB* SNPs in the Enhanced Resistance.

**Fig. 3.**
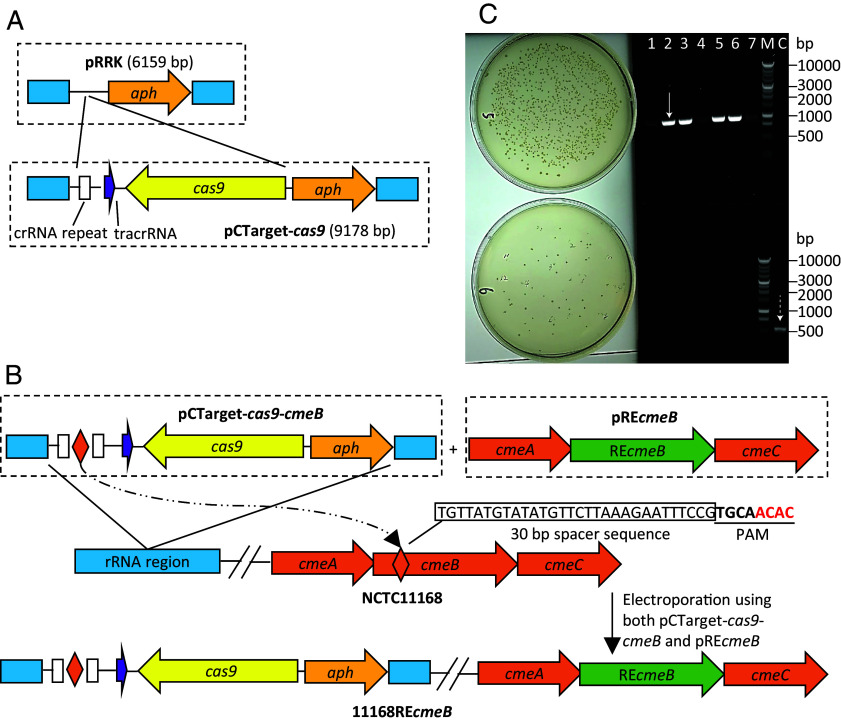
Overall strategy for targeted gene editing in *C. jejuni* using CRISPR-Cas9. (*A*) Construction of targeting plasmid pCTarget-cas9 using the pRRK plasmid. The solid blue boxes indicate rRNA sequences, while boxed arrows depict ORFs. The crRNA repeat is indicated by an open box. (*B*) The *cmeB*-specific targeting plasmid pCTarget-cas9*-cmeB* (dashed box on the *Left*) contains a 30 bp spacer sequence that is different from the corresponding RE-*cmeB* sequence, while the editing plasmid pRE*cmeB* (dashed box on the *Right*) contains a wild-type *cmeA* and *cmeC*, but the variant *cmeB* (RE-*cmeB*). pCTarget-cas9*-cmeB* and pRE*cmeB* are cotransferred into *C. jejuni* NCTC11168 by electroporation. The transformants were selected on MH agar plates containing 30 µg/ml kanamycin, which forces insertion of the target plasmid into the rRNA region via homologous recombination. The Cas9 recognizes the spacer sequence and kills the transformants that do not acquire RE-*cmeB*, but allows the growth of transformants with an acquired RE-*cmeB*. The combined actions generate unmarked gene editing in the *cmeABC* operon. Solid blue Boxes represent rRNA sequences, while boxed arrows represent different ORFs. Each open box represents a 36 bp crRNA repeat unit. The solid diamond represents a 30 bp *cmeB* unique spacer sequence for Cas9 recognition. The 30 bp *cmeB* spacer sequence along with the PAM sequence (5’-TGCAACAC-3’) is shown above the diamond. (*C*) Representative transformation results with different plasmids used in the CRISPR-Cas9-mediated gene replacement strategy. The *Left* panel shows kanamycin-resistant colonies from transformations using control plasmid pCTarget-cas9 and pREcmeB (*Top*) or pCTarget-cas9-cmeB and pREcmeB (*Bottom*). The control plasmid had no *cmeB*-specific spacer sequence. The *Right* panel shows PCR verification of seven transformants from the transformation using pCTarget-cas9-*cmeB* and pRE*cmeB*. The PCR was done using RE-*cmeB-*specific primers (REB-F/R) (*Top*) and *cmeB-*specific primers (11B-F/R) (*Bottom*). All primer sequences are listed in SI Appendix, Table S2. Lanes 1 to 7: PCR products of individual transformants; M, Promega 1 kb DNA ladder ladders; C, amplification from genomic DNA of NCTC11168 as a control. The arrow indicates RE-*cmeB-*specific product (933 bp), while the dashed arrow indicates *cmeB*-specific product (535 bp).

To verify the findings obtained from the high-throughput WGS analysis, we further generated a construct in *C. jejuni* NCTC11168, in which the original *cmeB* was replaced by RE-*cmeB* without replacing *cmeA* and *cmeC*. For this purpose, we developed a strategy utilizing the native CRISPR, CRISPR-Cas (CRISPR)-associated (Cas) system (CRISPR-Cas9) in *C. jejuni*. The targeting plasmid (Fig. 3*A*), named pCTarget-cas9 (9,178 bp), was constructed using the backbone of a suicide plasmid pRRK, which was designed to insert a gene into the rRNA region of *C. jejuni* (28). Plasmid pCTarget-cas9 contains a 3,269 bp sequence that encodes an intact *cas9* gene, tracrRNA, and one repeat unit from the crRNA region of NCTC11168. The reason for inserting only one crRNA repeat unit in pCTarget-cas9 was that a recent *C. jejuni* transcriptomic study found that each repeat unit in the *C. jejuni* crRNA region carries its own promoter (31). A 30-bp spacer sequence, which targets a region unique to *cmeB* (not RE-*cmeB*), and an additional copy of crRNA repeat were inserted into pCTarget-cas9, generating pCTarget-cas9-*cmeB* (Fig. 3*B*). In *cmeB*, this spacer sequence is followed by a PAM (5’-NNNNACAC-3’) (Fig. 3*B*), facilitating recognition by the Cas9 enzyme. The editing template containing a chimeric operon with RE-*cmeB* flanked by *cmeA* and *cmeC* of NCTC11168 was constructed using the backbone of pUC18 plasmid, generating an editing plasmid pRE*cmeB*. The targeting plasmid pCTarget-cas9-*cmeB* and the editing plasmid pRE*cmeB* were cotransferred to NCTC11168 by electroporation. A control reaction was carried out by cotransferring the empty targeting plasmid pCTarget-cas9 without a *cmeB* spacer sequence along with the editing plasmid pRE*cmeB* into NCTC11168 by electroporation. Insertion and expression of the *C. jejuni* Cas9 were expected to result in recognition of *cmeB* (guided by the *cmeB-*specific spacer sequence on the targeting plasmid) and killing of the bacterial cells carrying the native *cmeB* gene, which only allows growth of transformants with *cmeB* replaced by RE-*cmeB* via homologous recombination. Transformants were selected on kanamycin-containing plates. As shown in Fig. 3*C*, electroporation with targeting plasmid pCTarget-cas9-*cmeB* plus pRE*cmeB* yielded much fewer colonies than that with the empty targeting plasmid pCTarget-cas9 plus pRE*cmeB*, suggesting pCTarget-cas9-*cmeB* successfully targeted *cmeB* and elicited Cas9-mediated killing of kanamycin-resistant transformants in the absence of replacement by RE-*cmeB*. Verification by RE-*cmeB*-specific PCR amplification revealed that 4 out of the 7 randomly picked transformants from the transformation using the targeting plasmid pCTarget-cas9-*cmeB* carried RE-*cmeB* but not *cmeB* (Fig. 3*C*). The correct replacement of *cmeB* by RE-*cmeB* in the 4 transformants were further confirmed by DNA sequencing using primer walking. The remaining three transformants yielded a faint band with RE-*cmeB-*specific PCR and no bands with the *cmeB*-specific PCR (Fig. 3*C*). However, Sanger sequencing of the faint PCR products was not successful after repeated tries, with some sequencing primers did not yield readable sequence data, while other primers produced sequences that matched with both *cmeB* and RE-*cmeB* sequences, or even matched to the opposite strand, suggesting abnormal recombination happened in the *cmeB* locus of No. 1, 4, 7 transformants. Despite the fact that the correct replacement occurred only in 4/7 of the tested transformants, the results clearly indicated the high efficiency of the targeting plasmid pCTarget-cas9-*cmeB* in conjunction with the editing plasmid pRE*cmeB* in generating unmarked RE-*cmeB* replacement in *C. jejuni*. As a control for subsequent antibiotic susceptibility testing, a *C. jejuni* NCTC 11168 transformant generated by the empty targeting plasmid pCTarget-cas9 (lacking the 30 bp *cmeB* spacer sequence) was selected and named 11168_control_ (Table 1). MIC test results revealed that compared with 11168_control_, 11168REcmeB, in which *cmeB* was replaced with RE-*cmeB,* showed a 4-8-fold increase in the MICs of the tested antibiotics except for gentamicin (Table 3), confirming the key role of RE-*cmeB* mutations in the enhanced resistance.

**Table 3.** Antimicrobial MICs (µg/ml) of *C. jejuni* NCTC11168 and its mutant derivatives analyzed in this study

	NCTC11168	11168_control_		11168_A–G_		11168RE*cmeB*		11168_A–G_RE*cmeB*	
Antibiotics	MICs	MICs	Fold changes^*^	MICs	Fold changes^*^	MICs	Fold changes^*^	MICs	Fold changes^*^
Azithromycin	0.03	0.03	–	0.06	2	0.12	4	0.25	8
Clindamycin	0.06	0.06	–	0.25	4	0.25	4	0.5	8
Ciprofloxacin	0.06	0.06	–	0.12	2	0.25	4	0.5	8
Erythromycin	0.25	0.25	–	1	4	1	4	2	8
Florfenicol	0.5	0.5	–	1	2	4	8	8	16
Gentamicin	0.5	0.5	–	0.5	–	0.5	–	0.5	–
Nalidixic acid	≤4	≤4	–	8	≥2	8	≥2	16	≥4
Telithromycin	0.5	0.5	–	1	2	2	4	4	8
Tetracycline	0.12	0.12	–	0.5	4	0.5	4	1	8

^*^Fold increases compared with the *C. jejuni* wild-type strain NCTC11168. “–” indicated no change in MIC.

### Relative Contribution of Overexpression and Mutations of RE-CmeB to the Elevated Resistance to Antibiotics.

In order to dissect the specific contributions of the promoter mutation (resulting in overexpression) and the SNPs in RE-*cmeB* to the enhanced function of RE-CmeABC, a mutant strain (named 11168_A–G_) of NCTC11168 with the A–G mutation in the IR (Fig. 1*B*) was constructed by homologous recombination and selection on antibiotic plates (*Materials and Methods*). This promoter mutation is known to result in overexpression of *cmeABC* (21). To elucidate the combined effect of the promoter A–G mutation and the RE-*cmeB* SNPs, an unmarked RE-*cmeB* replacement in 11168_A–G_ strain was made utilizing the same CRIPSR-Cas9 method described above, generating *C. jejuni* mutant 11168_A–G_RE*cmeB* (Table 1). Then, various *C. jejuni* constructs were compared for antibiotic susceptibilities, including 11168_control_, 11168RE*cmeB*, 11168_A–G_, and 11168_A–G_RE*cmeB*. As shown in Table 3, the control strain 11168_control_ did not show any changes in the MICs of tested antibiotics compared to the wild-type NCTC11168. However, strain 11168_A–G_ showed a 2-4-fold increase, and 11168REcmeB demonstrated a 4-8-fold increase in antibiotic MIC except for gentamicin. Notably, 11168_A–G_RE*cmeB* showed the highest increase in the antibiotic MICs (4-16-fold increase except for gentamicin), demonstrating the additive effect of the promoter mutation and RE*cmeB* mutations on the enhanced resistance (Table 3). Importantly, the antibiotic MIC values for the 11168_A–G_RE*cmeB* strain were highly similar to or higher than those of the 11168*RE-cmeABC* transformants carrying the entire *RE-cmeABC* operon (Table 2), further indicating that the amino acid changes in RE-CmeA and RE-CmeC likely do not contribute to the enhanced function of RE-CmeABC in antibiotic resistance. The MIC results of these genetically defined constructs along with the WGS mapping data (Fig. 2) strongly indicated that the mutations in RE*-*CmeB and the A–G mutation in the CmeR binding site additively contribute to the enhanced function of RE-CmeABC, with the contribution of RE-*cmeB* greater than that of the A–G mutation in the *cmeA* promoter region.

### Distribution and Evolution of RE*-cmeABC* in *Campylobacter*.

**Fig. 4.**
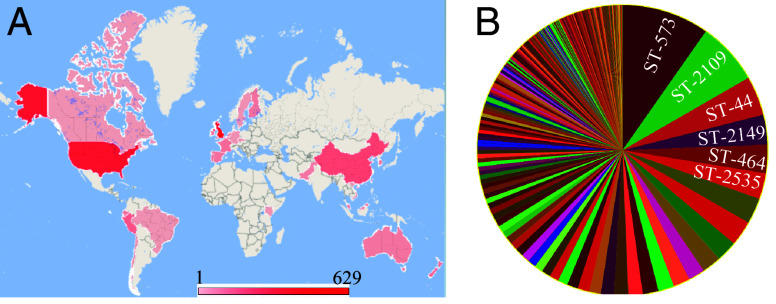
Global distribution and genetic diversity of 1,510 RE-*cmeB*-positive *Campylobacter* isolates identified in the NCBI Pathogen Detection database. (*A*) The geographic heatmap (Red) shows the global distribution of RE-*cmeB*-positive *C. jejuni* and *C. coli.* The scale bar indicates the number of isolates from countries. (*B*) The percentage of sequence types of RE-*cmeB*-positive *Campylobacter* isolates. Six top STs from the 214 known STs are labeled.

RE*-cmeABC* was first reported in 2016 (21). To assess its evolution and spread, we analyzed *Campylobacter* genomes deposited in the NCBI Pathogen Detection database. Among the 57,072 *C. jejuni* and 25,299 *C. coli* genomic sequences that were deposited in the database as of May 10, 2023, 1510 isolates carried RE*-cmeABC*, including 32 *C. coli* and 1478 *C. jejuni*. This result indicates that RE-*cmeABC* is primarily associated with *C. jejuni*. Based on the sequences in the database, the earliest detection of RE-*cmeABC* was 1996 in *C. jejuni* and 2010 in *C. coli*, and there were only 39 pre-1996 *Campylobacter* isolates in the database. The RE-*cmeABC*-positive isolates were sourced from 25 countries across all the continents, and mostly from the United Kingdom (41.7%; n = 629) and United States (25.4%; n = 384) (Fig. 4*A* and Dataset S3). They were from multiple host species including humans, dogs, cats, chickens, ducks, turkey, geese, sheep, cattle, pigs, monkeys, and retail meat (Dataset S3); however, most of them (68.7%; n = 1038) of them were associated with human cases. Sequence typing showed that RE-*cmeABC* was found in genetically diverse strains, represented by 214 known sequence types (STs) and 432 isolates with unassigned STs (SI Appendix, Fig. S2 and Dataset S3). The wide distribution of RE-*cmeABC* in different genotypes and even different species indicates that horizontal gene transfer played a fundamental role in its spread. Among the known STs, ST573 and ST2109 of *C. jejuni* were the main sequence types (Fig. 4*B* and Dataset S3). ST573 was primarily from the United Kingdom (104/105), while ST2109 was only found in the United States.

Notably, *C. jejuni* ST2109 represents an extensively drug-resistant lineage and has been implicated in recent outbreaks of human campylobacteriosis associated with exposure to puppies (24, 32). All ST2109 isolates carried multiple (≥3) antibiotic resistance genes/determinants in addition to RE*-cmeB* (Dataset S3). Antimicrobial susceptibility testing of ten selected RE*-cmeB*-positive *C. jejuni* ST2109 isolates showed that they were extensively resistant to the tested antibiotics including macrolides, fluoroquinolones, aminoglycosides, lincosamides, and tetracyclines (SI Appendix, Table S1), consistent with the previously reported results (32) and the findings of genome-based analysis (Dataset S3). The high-level resistance to multiple antibiotics in ST-2109 is likely due to the combinatorial effect of the acquired RE*-cmeB* and other antibiotic resistance mutations/genes, such as the A2035G mutation in the 23S rRNA gene, T86I mutation in GyrA, aminoglycoside resistance genes [e.g., *aad9*, *aadE*, *aph(2'')-Ih*, *aph(3')-IIIa*], and *tet*(O) gene conferring resistance to macrolides, quinolones, gentamycin, and tetracycline, respectively. Interestingly, different from other RE-*cmeB-*positive isolates carrying the A–G promoter mutation, all the examined RE-*cmeB*-positive *C. jejuni* ST2109 isolates carried a C-T mutation in the CmeR binding site in the promoter region, which was reported to be associated with CmeABC overexpression and enhanced resistance to antimicrobials (33). Together, the results indicate that RE-*cmeB*-positive *C. jejuni* is expanding globally and has developed resistance to multiple antibiotics, with some genotypes (e.g., ST-2109) implicated in disease outbreaks.

**Fig. 5.**
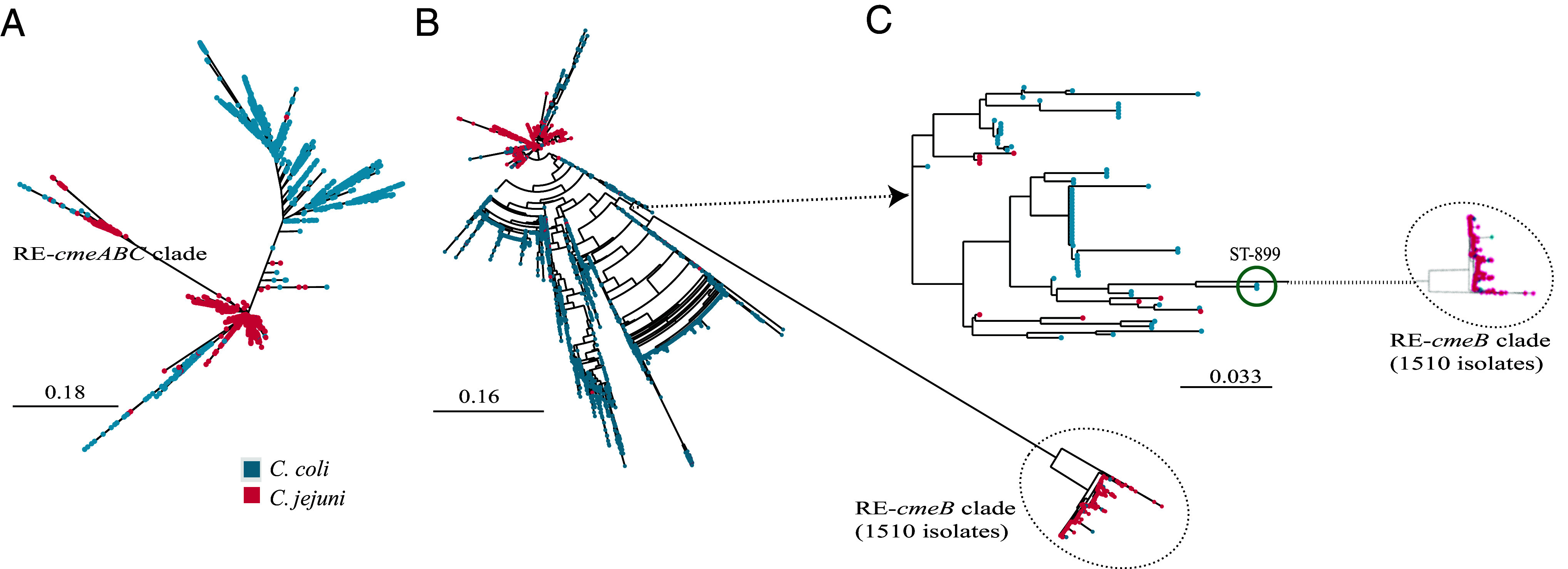
Phylogenetic analysis of RE-*cmeABC* from 57,072 *C. jejuni* and 25,299 *C. coli* in the pathogen detection database. Green: *C. coli*; Red: *C. jejuni*. Scale bar, nucleotide substitutions per site. (*A*) Phylogenetic analysis of 8,133 different *cmeABC* sequence types. The RE-*cmeABC* clade is composed of 463 RE-*cmeABC* sequence types from 1,510 *C. jejuni* and *C. coli* isolates and is separated from other *cmeABC* clades in both species. (*B*) Phylogenetic analysis of *cmeB* sequences. The RE-*cmeB* clade including 1,510 *C. jejuni* and *C. coli* isolates originated from *C. coli*. (*C*) Zoom-in view of 76 isolates with closely related *cmeB* sequences suggests that the RE-*cmeB* clade may have evolved from *C. coli* ST-899.

From the 82,371 *C. jejuni* and *C. coli* genomic sequences deposited in the pathogen detection database, 8,133 different *cmeABC* sequence types were identified. Phylogenetic analysis of these *cmeABC* sequence types revealed that RE-*cmeABC* formed a distinct clade (Fig. 5*A*). RE-*cmeABC* (GenBank: KT778507.1) has 16 SNPs in RE-*cmeA*, 595 SNPs in RE-*cmeB*, and 201 SNPs in RE-*cmeC* compared to the typical *cmeABC* in *C. jejuni* NCTC 11168. However, not all the SNPs were present in every isolate in the RE-*cmeABC* clade, and the 1510 RE-*cmeABC*-positive isolates of the clade yielded 463 different RE*-cmeABC* sequence types (Fig. 5*A*), suggesting that the clade was developed in an evolutionary process. Given the importance of RE-*CmeB* in the enhanced function in antibiotic resistance, phylogenetic analysis was further done using the *cmeB* sequences from the 57,072 *C. jejuni* and 25,299 *C. coli* isolates in the database, which showed a distinct RE-*cmeB* clade far separated from other *cmeB* clades (Fig. 5*B*). Although RE-*cmeB* is predominantly distributed in *C. jejuni*, it appears that it originated from *C. coli* (Fig. 5 *B* and *C*). The closest relative of RE-*cmeB* in the tree was *C. coli* ST899 (Fig. 5*C*), but detailed amino acid sequence alignment of RE-CmeB with the CmeB in ST899 indicated that they remain divergent (SI Appendix, Fig. S3). Thus, the intermediate sequences between the CmeB of ST899 and RE-CmeB remain to be determined.

**Fig. 6.**
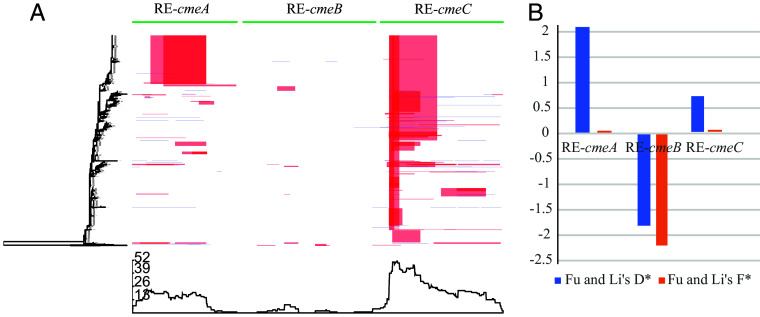
Recombination and positive selection analysis of RE-*cmeABC*. (*A*) Recombination landscape of RE-*cmeABC* from 1,510 *C. jejuni* and *C. coli* genomes. Left: Maximum likelihood phylogeny generated from the nonrecombinant regions of RE-*cmeABC* of the 1,510 isolates. Top: RE-*cmeA*, RE-*cmeB*, and RE-*cmeC* annotation along the length. Middle: Distribution of recombination events across the 1,510 *Campylobacter* isolates. Each row represents an isolate and the columns relate to bases in RE-*cmeABC*. The red columns are recombinations shared by multiple isolates and occurring in the internal branches. The light blue columns are recombinations in the terminal branch and represented by unique isolates. Bottom: The line graph represents the frequency of recombination events along RE-*cmeABC* region. (*B*) Positive selection analysis by Neutrality tests. Fu and Li’s D* (blue) and Fu and Li’s F* (orange) tests show that RE-*cmeB* experienced selective sweeps (negative values) but not RE-*cmeA* and RE-*cmeC* (positive values), which is supported by coalescent Simulation (10,000 sampling replicates) with Fu and Li’s D* (*P* = 0.0199) and Fu and Li’s F* (*P* =0.0053) and MDK test (RE-*cmeA P* > 0.05, RE-*cmeB P* < 0.001, and RE-*cmeC P* > 0.05). The values on the Y-axis show the selection value generated by Fu and Li’s D* test and Fu and Li’s F* test for RE-*cmeA*, RE-*cmeB,* and RE-*cmeC,* respectively.

In *Campylobacter*, recombination is a powerful evolutionary force, often leading to the emergence of new alleles of genes, new lineages, or even large-scale genome-wide intra- and interspecies introgression, especially between *C. jejuni* and *C. coli* (34, 35). To further understand the evolution of RE-*cmeABC*, we performed recombination analysis using Gubbins (36). As shown in Fig. 6*A*, recombination was detected frequently in RE*-cmeA* and RE*-cmeC*, but rarely in RE*-cmeB*, even though the three genes are genetically linked, i.e., RE*-cmeB* being localized in the middle of *the* RE-*cmeABC* operon. The finding suggests that the linkage disequilibrium of RE-*cmeA*, RE-*cmeB*, and RE-*cmeC* was interrupted during evolution. Given that CmeABC is associated with resistance to antibiotics and hence facilitate the survival of *Campylobacter* under selection pressure, positive selection may have influenced the linkage disequilibrium of the three genes and created a less variable RE*-cmeB* by selective sweeps that increase the favored alleles and diminish the neighboring linked variations in the population (37, 38). Thus, the difference in recombination frequencies suggests that RE-*cmeB*, but not RE-*cmeA* and RE-*cme*C, experienced selective sweeps and was positively selected during evolution and adaptation. To test this hypothesis, population genetics methods (Fu&Li’s D*, Fu&Li’s F*, and MDK test), which were known to be sensitive to detect ongoing or relatively recent sweep events (37, 38), were applied to detect the positive selection signals in RE*-cmeABC*. All three tests indicated that RE*-cmeB,* but not RE-*cmeA* and RE-*cmeC*, experienced a selective sweep (Fig. 6*B*) with a statistically significant *P-*value (*P* = 0.0199 for Fu and Li’s D*, *P* = 0.0053 for Fu and Li’s F*, and *P* < 0.001 for MDK test). Notably, the most significantly selected amino acids predicted by Fu & Li’s D* and Fu & Li’s F* tests are located in the drug-binding pocket (SI Appendix, Fig. S4) of RE-CmeB as identified by the structural studies recently (39). Together, the results from the evolutionary analysis corroborate the conclusion that mutations in RE-CmeB are important for the enhanced antibiotic resistance function and are progressing to be fixed during the evolutionary process.

## Discussion

In this study, we demonstrated that SNPs in RE-*cmeB*, but not those in RE-*cmeA* and RE-*cmeC*, along with a mutation in the promoter region that results in overexpression of the efflux operon are necessary for the enhanced function of RE-CmeABC (Figs. 1–3 and Table 3), revealing the genetic basis for the enhanced function of RE-CmeABC in antibiotic resistance of *Campylobacter.* We also showed the feasibility of using CRISPR-Cas9 for gene replacement in *Campylobacter.* Additionally, the results revealed a worldwide distribution of RE-CmeABC in genetically diverse *Campylobacter* strains including *C. jejuni* strains implicated in disease outbreaks. Phylogenetic analysis and recombination analysis suggested that RE-CmeB originated from *C. coli* and experienced positive selection and fixation during evolution. Given its important role in antibiotic resistance, fixation of RE-CmeB may have provided an advantage for *Campylobacter* adaptation to antibiotic selection pressure.

The high-throughput mapping strategy effectively identified function-conferring mutations in RE-*cmeB* and the IR of the promoter region (Fig. 2). This method is sensitive to detect allele frequency changes at a single nucleotide level, as demonstrated by the 99.1% transfer frequency of the A–G mutation in the IR to the wild-type NCTC11168 background as opposed to the very low transfer frequency (3.2%) of the same mutation to the 11168Δ*cmeR* background (Fig. 2 *A* and *B*). This can be explained by the fact the A–G mutation prohibits the binding of the CmeR repressor to the *cmeABC* promoter and therefore leads to overexpression of the efflux transporter, while in the 11168∆*cmeR* background, the efflux system is constitutively overexpressed and the promoter mutation is no longer needed for overexpression of *cmeABC* (20). Overexpression of the efflux pump is partly needed for the enhanced resistance to antibiotics and thus a promoter mutation was nearly unanimously present in the florfenicol-resistant transformants generated from the wild-type 11168 background. The accuracy and sensitivity of the high-throughput mapping strategy make it useful for screening function-conferring mutations in *Campylobacter* and potentially in other bacterial species.

Compared to the typical CmeB in *C. jejuni* NCTC11168, RE-CmeB harbored 198 amino acid substitutions (21), among which 75 were uniformly transferred to the florfenicol-resistant transformants (SI Appendix, Fig. S1 and Dataset S1), suggesting their important role in antibiotic efflux. According to the crystal and cryoelectron microscopy (cryo-EM) structures of *C. jejuni* CmeB and RE-CmeB (39, 40), most of these amino acids are located in the periplasmic domain formed by two extracellular loops between transmembrane helices TM1 and TM2, and between TM7 and TM8, respectively (SI Appendix, Fig. S1). This large periplasmic domain contains 6 subdomains and forms a large internal cavity constituting multiple drug-binding sites, which include the entrance, proximal, and distal sites, playing a predominant role in substrate recognition and binding (39, 40). Previously, molecular structure modeling predicted several mutated amino acids of RE-CmeB including I136, V139, I291, V605, and L607 were more likely to be used for RE-CmeB to bind drug molecules such as ciprofloxacin and florfenicol (21). The cryo-EM structures of RE-CmeB both in the absence and presence of ciprofloxacin, chloramphenicol, erythromycin, or hydrolyzed ampicillin revealed many of these bound drug molecules were observed to span the proximal and distal multidrug-binding site of the periplasmic domain of RE-CmeB, where these four drug-binding sites are partially overlapped with each other (39). The structures also illustrated that the RE-CmeB transporter utilizes slightly different subsets of residues to bind these drugs compared to CmeB, thus optimizing the capacity of recognizing and extruding a broad spectrum of distinct classes of antibiotics. Based on these cryo-EM structures, it appears that RE-CmeB frequently uses I136, V139, I291, Y328, M570, F610, L612, F613, F625, and L662 to bind these drugs, among which Y328, F610, L612, F613, F625, and L662 are conserved between CmeB and RE-CmeB (SI Appendix, Fig. S1). It should be noted among these identified residues important for drug binding, I291, M570, and V605 were uniformly transferred to NCTC 11168 and 11168Δ*cmeR* transformants. In addition, I136 and V139 were uniformly present in the NCTC11168 transformants (>99.5% transfer frequency) and were detected in the vast majority of the 11168Δ*cmeR* transformants with frequencies of 98.3% and 98.4%, respectively (Dataset S1). Similarly, L607 was uniformly transferred into the 11168Δ*cmeR* transformants (>99.5% transfer frequency) and was detected in the vast majority (98.2%) of the 11168 transformants (Dataset S1). These results suggest that amino acid mutations in the drug-binding sites may play an important role in the enhanced function. Although the mutations in RE-CmeB enhanced its function in antibiotic extrusion, it did not seem to alter the substrate specificities compared to the wild-type CmeB (Table 2). However, only a limited number of antibiotics were examined in this study, which does not exclude the possibility that RE-CmeB may recognize additional antimicrobials. Notably, the MIC increase for florfenicol was the most among the tested antibiotics (Table 2), suggesting RE-CmeB has a particular preference for extruding this antibiotic.

Previous studies demonstrated that the CmeABC proteins were posttranslationally modified by N-glycosylation, which enhanced the function of CmeABC in antibiotic efflux (41, 42). Interestingly, the known glycosylation site in CmeB (N636) was mutated to E635 in RE-CmeB (SI Appendix, Fig. S1), suggesting that the protein lost this posttranslational modification. Given that E635 was one of the amino acids unanimously transferred to the gain-of-function transformants (SI Appendix, Fig. S1 and Dataset S1) and RE-CmeB showed an enhanced antibiotic resistance phenotype, it appeared that loss of the glycosylation site did not negatively impact the function of RE-CmeB. Another interesting observation is that the transferred amino acid substitutions are distributed across the entire CmeB sequence and are not just localized in one specific region (SI Appendix, Fig. S1 and Dataset S1), which suggests that multiple amino acid changes across different parts of the RE-CmeB transporter are required for its full functional gain. This is in contrast to previously reported gain-of-function mutations in RND-type transporters of other bacterial species, where a single or a few amino acid changes were responsible for the functional gain in antibiotic resistance (9, 10). However, it should be pointed out that not all of the transferred amino acid substitutions are necessary for the enhanced function. Some of the detected transfers may arise from their close proximity to functionally essential residues and consequently are cotransferred during the homologous recombination. This cotransferring effect was also seen with the SNPs in RE-*cmeA* and RE-*cmeC*. Although SNPs in these two genes do not contribute to the functional gain, the transfer frequencies of the SNPs showed a decreasing trend along the lengths of *cmeA* and *CmeC* moving away from *cmeB* (Fig. 2 *A* and *B*). The same effect was not observed with the transfer of SNPs in RE-*cmeB*, further suggesting that amino acid substitutions across multiple regions of RE-CmeB are likely required for its enhanced antibiotic resistance function.

A bottleneck in genetic modifications of *Campylobacter* is the generation of unmarked gene replacement or mutations in a gene of interest. It remains difficult and inefficient despite the availability of a previously reported method that utilizes a *cat*-*rpsL* cassette and two steps of negative antibiotic selection (43, 44). Although the method has been successfully used in several studies (44, 45), failed attempts were also reported (46). The recent discovery of CRISPR-Cas systems, which are prokaryotic adaptive immune systems against invading mobile elements (e.g., phages and plasmids) (47, 48), provides a potentially effective way for gene editing in bacterial species. The relatively simple design and the ability to select for an unmarked mutation without introducing a scar site in the target gene make genome editing with CRISPR advantageous over other methods. Recently, a type II CRISPR-Cas9 system of *Streptococcus pyogenes* was successfully engineered for genome editing (49). In this system, Cas9 nuclease from *S. pyogenes* (SpCas9) is directed by guide RNAs (gRNAs) to cleave complementary DNA sequences flanked by a specific protospacer adjacent motif (PAM) (48). So far, the *S. pyogenes* CRISPR-Cas9 system has been successfully applied for gene editing in a few bacterial species, in which a native CRISPR-Cas system is absent (50–54). In *Campylobacter*, a minimal type II CRISPR-Cas9 system including an actively transcribed CRISPR-RNA (crRNA) and a transactivating crRNA (tracrRNA) was identified by RNA-seq analysis of *C. jejuni* isolates (31). Recently, Song et al. (55) determined the PAM sequences (5’-NNNNACAC-3’ or 5’-NNNNRYAC-3’) for *C. jejuni* Cas9 (CjCas9) and successfully introduced target mutations in mouse muscle cells and retinal pigment epithelium cells by using CjCas9-mediated gene editing. In this study, we successfully used the native CRISPR-Cas9 system and the 5’-NNNNACAC-3’ PAM sequence for gene editing in *Campylobacter*. The successful replacement of *cmeB* by RE-*cmeB* was confirmed by both PCR amplification (Fig. 3*C*) and DNA sequencing as well as phenotypic analysis of the mutant constructs (Table 3). The editing method did not involve insertion of an antibiotic resistance marker within or nearby the *cmeB* gene, although the *aph* gene (encoding kanamycin resistance) was used to insert the target plasmid into the chromosome (Fig. 3*B*). Additionally, the CRISPR-Cas9-mediated editing only involves a single step of positive selection, which is simpler than the *cat*-*rpsL* based method. Furthermore, a targeting plasmid that is specific for a gene of interest can be constructed using pCTarget-cas9 by simply modifying the 13 bp of 5’ sequences of primer cmeB-sp-F and the 17 bp of 5’ sequences of primer cmeB-sp-R to replace the spacer sequence (SI Appendix, Table S2). However, this gene editing method requires a PAM sequence nearby the spacer sequence (Fig. 3*B*), which may not be available and hence limits its use for mutations in certain target genes.

Analysis of the *C. jejuni* genomic sequences deposited in the Pathogen Detection Database clearly revealed the emergence of RE*-cmeABC* harboring isolates on a global scale (Fig. 4*A*). Our finding is consistent with a recent report by Yao *et al.* who reported 433 RE*-cmeABC*-positive isolates from different countries (30). The increased prevalence of RE-CmeB was also shown in recent publications from different geographical regions, where up to 70.7% *C. jejuni* isolates from chickens and 74.3% *C. jejuni* isolates from human campylobacteriosis cases were found to carry RE-*cmeABC* (21–23). The continued spread of RE-*cmeABC* in *Campylobacter* populations could be explained by its fitness advantage under antibiotic selection pressure as possession of this variant efflux transporter enables *C. jejuni* to be more resistant to antibiotics. Based on the distribution analysis, RE-*cmeABC*-positive isolates were associated with a variety of animal hosts (Dataset S3), suggesting that this antibiotic efflux pump facilitates the adaptation of *Campylobacter* in diverse host environments. Despite the fact that RE*-cmeABC* was first reported and highly prevalent in China (21, 22), the majority of the RE*-cmeB*-positive strains in the NCBI Pathogen Detection database were from the United Kingdom and the United States (Fig. 4*A* and Dataset S3). One possible explanation for this discrepancy is that the *C. jejuni* whole genome sequences in the NCBI Pathogen Detection database overrepresented the countries that actively conduct whole genome sequencing of *C. jejuni* and submit the information to the NCBI Pathogen Detection database. With the continued increase of *Campylobacter* genome sequences deposited in the database, it is likely that more RE*-cmeABC* harboring isolates will be identified around the world.

Phylogenetically, RE*-cmeAB*C forms a unique clade but is present in genetically diverse *C. jejuni* strains, suggesting that this variant efflux transporter may spread via horizontal gene transfer (Fig. 5*A* and SI Appendix, Fig. S2 and Dataset S3). This possibility is supported by the observation under laboratory conditions, where RE-*cmeABC* can be transferred across different *Campylobacter* species and strains (21). Interestingly, predominant genotypes that carry RE-*cmeB*, such as ST-573 and ST-2109, were primarily observed in certain geographical regions (Fig. 4 and Dataset S3). For example, ST-573 represents a major *C. jejuni* genotype isolated from poultry in the United Kingdom (56), while RE*-cmeABC*-positive ST-2109 *C. jejuni* isolates were exclusively found in the United States. This suggests regional expansion occurred with RE*-cmeABC* harboring clones. In addition to RE*-cmeB*, ST-2109 isolates carried other antibiotic resistance determinants (Dataset S3), and the joint function of these mechanisms essentially makes them resistant to most clinically important antibiotics, as reported previously (32) and confirmed by the MIC results in this study (SI Appendix, Table S1). Importantly, ST-2109 isolates have been implicated in a large outbreak of human campylobacteriosis associated with exposure to pet store puppies in multiple States in the United States (32). Compared to other ST types, ST-2109 is a rare genotype and is mostly associated with dogs. How ST-2109 emerged in dogs and how it acquired RE*-cmeB* remain unclear and warrant further investigation.

Based on phylogenetic analysis, it appeared that RE-*cmeB* originated from *C. coli* but remained distant from its closest relative *C. coli* ST899 (Fig. 5 and SI Appendix, Fig. S3), suggesting a rapid diversification from its ancestral allele and fast expansion in *Campylobacter* populations, particularly in *C. jejuni*. Interestingly, intermediate sequences between the *cmeB* of ST899 and RE-*cmeB* were not identified in the *Campylobacter* genome sequences deposited in the NCBI database. Thus, there remains a possibility that RE-*cmeB* was generated by an event of horizontal gene transfer and recombination. Regardless of the origin of RE-*cmeB*, its rapid expansion in *C. jejuni* is likely driven by antibiotic usage in both human medicine and animal husbandry as it makes *Campylobacter* more resistant to antibiotics and may confer *Campylobacter* a fitness advantage under antibiotic selection pressure. Interestingly, sequence analysis frequently detected recombination in RE-*cmeA* and RE-*cmeC* but not in the RE-*cmeB* allele (Fig. 6*A*). Similarly, positive selection analysis demonstrated that RE-*cmeB*, but not RE-*cmeA* and RE-cmeC, experienced selection sweeps (Fig. 6*B*) and the signal was particularly strong surrounding the region that is involved in the interaction with drugs (SI Appendix, Figs. S1 snd S3). Additionally, Weblogo alignment of RE-CmeB sequences from the 1,510 isolates revealed little variation, indicating the highly conserved nature of the RE-*cmeB* allele (SI Appendix, Fig. S5). These results from sequence analysis are consistent with the experimental findings that the amino acid changes in RE-CmeB are important for the functional gain in antibiotic resistance, while the mutations RE-CmeA and RE-CmeC are dispensable for the functional change. Together, these results strongly suggest that once emerged, the RE-*cmeB* allele expanded rapidly in *C. jejuni* and was progressing to be fixed during evolution, probably due to its key function in shaping *Campylobacter* adaptation to antibiotic selection.

In summary, findings from this study identified the molecular mechanisms underlying the enhanced antibiotic resistance function of RE-CmeABC in *Campylobacter*, which is mediated by the mutations in the RE-*cmeB* gene in conjunction with a mutated IR in the promoter region of the efflux operon. The amino acid substitutions in RE-CmeB likely influence its interaction with antibiotic and/or the efflux function of the pump, while the IR mutation enhances the expression of the antibiotic efflux system. The joint action results in simultaneously enhanced resistance to multiple drugs, which represents a distinct mechanism for clinically relevant antibiotic resistance mediated by an RND efflux transporter in gram-negative bacteria. Given the importance of RE-CmeABC in antibiotic resistance, its presence may be considered as a marker for genome-based surveillance of antibiotic-resistant *Campylobacter*. This is especially possible for florfenicol as RE-*cmeABC* alone is able to raise the MIC to ≥ 4 µg/ml (Table 2) (21), which is the epidemiological cutoff value established by the European Committee on Antimicrobial Susceptibility Testing (57). RE-*cmeABC* alone may not be sufficient to predict a multidrug resistance phenotype, but coexistence of this efflux pump variant with other antimicrobial resistance mechanisms may serve as an indicator for high-risk *Campylobacter* strains. Additionally, our study found global spread of this potent efflux system in *C. jejuni* and revealed that RE-c*meB* likely originated from *C. coli* and its evolution is likely driven by antibiotic usage. These findings define how RE-CmeABC contributes to clinically relevant antibiotic resistance and shed light on how it has facilitated *Campylobacter* adaption to antibiotic selection in various host species. Furthermore, the study developed a CRISPR-Cas9-mediated gene editing method for efficient gene replacement in *C. jejuni*, which may be adapted as a tool to generate unmarked in-frame deletions or mutations for functional characterization of various genes in *Campylobacter* and other bacterial species.

## Materials and Methods

### Bacterial Strains and Culture Conditions.

*C. jejuni* NCTC11168 (26) and its derivatives used in this study and their sources are listed in Table 1. Additionally, 10 *C. jejuni* isolates of ST-2109 used for antimicrobial susceptibility testing (AST) to determine the MICs were obtained from the US National Antimicrobial Resistance Monitoring System. Bacterial cultures were grown in Mueller-Hinton (MH) broth or on MH agar at 42 °C under microaerobic conditions (5% O_2_, 10% CO_2_, and 85% N_2_).

### Transformation of RE-*cmeABC* into *C. jejuni* NCTC11168.

Electroporation was conducted as described previously (58). Briefly, a PCR product containing the whole RE-*cmeABC* operon, its promoter region, and flanking sequences on both ends was amplified from the genomic DNA of *C. coli* DH161 where RE-*cmeABC* was originally detected (GenBank accession number: KT778508.1) (21) using primer pairs REcmeABC-F/R (SI Appendix, Table S2). The amplified RE-*cmeABC* PCR fragment served as the donor DNA and *C. jejuni* NCTC11168 was used as the recipient strain. The transformants were selected on MH agar plates containing florfenicol (4 µg/ml). PCR and DNA sequencing were performed to confirm the presence of RE-*cmeABC* in the transformants.

### AST.

Commercially available Sensititre *Campylobacter* plates (Thermo Fisher Scientific, Waltham, MA) containing nine antimicrobials were used for AST of *C. jejuni* strains carrying various constructs. The plates were read after incubation under microaerobic conditions for 24 h at 42 °C. The lowest antimicrobial concentration at which no bacterial growth observed was used as the MIC for each isolate.

### Mapping of the Regions of RE-*cmeABC* Essential for Enhanced Function in Antibiotic Resistance.

To identify the mutations required for the enhanced function in individual gain-of-function RE*cmeABC* transformants, we developed a high-throughput method utilizing natural transformation, positive selection, and whole genome sequence analysis. For this purpose, the RE-*cmeABC* PCR fragment was electroporated into either wild-type *C. jejuni* NCTC11168 or its 11168Δ*cmeR* strain, in which the *cmeR* repressor gene for the *cmeABC* operon was deleted and thus the efflux operon is constitutively overexpressed (20). Transformants with enhanced antibiotic resistance were selected on MH agar plates containing 4 µg/ml florfenicol. This antibiotic concentration is 8 times higher than the MIC of NCTC 11168 with a typical CmeABC (Table 2) and was chosen because a previous study found that most RE-*cmeABC* harboring isolates had a florfenicol MIC of ≥4 µg/ml (21). In total, 273 colonies from the NCTC11168 transformation and 550 colonies from the 11168Δ*cmeR* transformation were randomly picked and subcultured onto new florfenicol containing MH plates to ensure the purity of the transformants. After an overnight growth, the subpassaged RE-*cmeABC* containing transformants were washed off the plates and pooled for subsequent genomic DNA extraction, which was conducted using a Wizard Genomic DNA Purification Kit (Promega) according to the manufacturer’s instructions. About 4 µg of purified genomic DNA was submitted for library preparation using TruSeq DNA PCR-Free Library Prep Kits (Illumina). An Illumina MiSeq system was used to sequence the prepared genomic DNA library with read lengths of 2 × 150 bp. After sequencing, quality control of the raw sequence reads was done by using FastQC (https://www.bioinformatics.babraham.ac.uk/projects/fastqc/) and trimmed by Trimmomatic (59). The trimmed reads were mapped against *C. jejuni* NCTC11168 genome sequences using Bowtie2 (60). Allele frequencies of the mapped reads were analyzed by LoFreq (61). The reason for using PCR-Free Illumina Library Prep Kits and whole genome sequencing instead of amplicon sequencing of the PCR product of the *cmeABC* region of the pooled transformants was to avoid any bias or spontaneous mutations potentially introduced during the PCR amplification.

### Generation of Various Plasmid Constructs for Making Mutations in *Campylobacter*.

All PCR primers used in this study are listed in SI Appendix, Table S2. The suicide plasmid pRRK (28) was linearized by using primer pairs pRRK-lF/lR and inverse PCR. The CRISPR-Cas region (4,576 bp) of *C. jejuni* NCTC11168 containing *cas9*, *cas1*, *cas2*, tracrRNA, and one repeat unit of the crRNA sequences was amplified by using primer pairs cjCRISPR-F/R. The linearized pRRK plasmid was ligated with the CRISPR-Cas fragment utilizing the SLiCE cloning method (62) to construct plasmid pRRK-cjCRISPR (10,485 bp). The targeting plasmid pCTarget-cas9 (Fig. 3*A*) was constructed from the pRRK-cjCRISPR plasmid by using inverse PCR and ligation methods. Briefly, an inverse PCR primer pair pCTarget-cas9-F/R was used to linearize pRRK-cjCRISPR plasmid and delete the *cas1*, *cas2* sequences. The linearized pRRK-cjCRISPR fragment with deletion was treated by DpnI enzyme (New England BioLabs) to remove remnant template plasmid DNA, pRRK-cjCRISPR, used for PCR amplification. DpnI treated PCR fragment was then phosphorylated using T4 Polynucleotide Kinase (New England BioLabs) and self-ligated by using the Quick Ligation Kit (New England BioLabs) to construct the pCTarget-cas9 plasmid. To insert the *cmeB* spacer sequence (30 bp) and another crRNA repeat unit (36 bp) for CjCas9 recognition, a pair of primer cmeB-sp-F/R was designed so that cmeB-sp-F contained 13 bp from 3’ end of the *cmeB* spacer sequences and cmeB-sp-R contained 17 bp from the reverse complement of 5’ end of the *cmeB* spacer sequences. The inverse PCR strategy described above was used to amplify the pCTarget-cas9 plasmid (Fig. 3*B*). To construct the editing plasmid pRE*cmeB* (Fig. 3*B*), four PCR fragments including *cmeA*, RE-*cmeB*, *cmeC,* and linearized pUC18 (27) were amplified by primer pairs cmeA-F/R, REcmeB-F/R, cmeC-F/R, and pUC18-lF/lR, respectively, and then ligated utilizing the SLiCE cloning method as described above. To generate a 11168_A–G_ mutant strain containing the A–G mutation in the *cmeA* promoter region, a plasmid pCmeAP_A–G_ with the A–G mutation was constructed using the pUC18 backbone. For this purpose, the flanking region of the A site to be mutated in the *cmeA* promoter region was amplified from NCTC11168 strain using primers cmeAP-F/R and ligated with a linearized pUC18 fragment by using pUC18-lF1/lR1 primers and the SLiCE cloning method as described above, generating plasmid pCmeAP. The A–G mutation was introduced into pCmeAP by using the same inverse PCR method as mentioned above using primers cmeAPG-F/R, yielding plasmid pCmeAP_A–G_.

## Introduction of the A–G Mutation in the *cmeA* Promoter into *C. jejuni* NCTC11168.

In the promoter region of *cmeABC*, there is an inverted repeat that serves as the binding site for transcriptional repressor CmeR, and alteration of the IR prohibits CmeR binding and results in overexpression of the *cmeABC* (20). Overexpression *cmeABC* leads to modest (2-4-fold) MIC increases of several antibiotics including erythromycin and clindamycin (20, 63). This feature allowed us to select the A–G substitution in the CmeR-binding site (Fig. 1*B*) using transformation and antibiotic selection. For this purpose, plasmid pCmeAP_A–G_ containing the A–G substitution in the *cmeABC* promoter was electroporated into NCTC11168. The 11168_A–G_ transformants were selected on MH agar plates containing 0.5 µg/ml of erythromycin and 0.15 µg/ml of clindamycin. The desired A–G mutation in the *cme*ABC promoter of the transformants was confirmed by PCR and DNA sequencing.

### CRISPR-Cas9-Mediated RE-*cmeB* Replacement in *C. jejuni* NCTC11168.

To construct 11168RE-*cmeB* and 11168_A–G_RE*cmeB* mutants, in which the native *cmeB* of 11168 was replaced by RE-*cmeB*, electroporation was conducted as previously described (64) with some modifications. To prepare the electrocompetent cells, *C. jejuni* was cultured overnight at 42 °C on a fresh MH agar plate under microaerobic condition for 14 to 16 h. Cells were harvested using 1 ml fresh MH broth into a 1.5 ml Eppendorf tube and then centrifuged for 5 min at 5,000 rpm at 4 °C (approximately 3000×*g*). The cell pellet was then gently resuspended in 1 ml of ice-cold wash buffer (272 mM sucrose, 15% glycerol) and centrifuged. The wash step was repeated for three more times. The final washed pellet was resuspended in 1 ml of ice-cold wash buffer. The competent cells were divided into 100 µl aliquots and kept on ice. For electroporation, a 1 mm electroporation cuvette (Bio-Rad) was prechilled on ice. A total amount of 4 µg DNA including 2 µg of targeting plasmid pCTarget-cas9-*cmeB* and 2 µg of editing plasmid pRE*cmeB* were added into the competent cells and gently mixed by pipetting. The total volume of DNA added was less than 10 µl to avoid sparking during electroporation. A total volume of 80 µl of cells-DNA mixture was added into the prechilled cuvette carefully to avoid formation of bubbles. Electroporation was carried out with the following configurations: 2.5 kV, 200 Ohms, and 25 µF (time constant should be >4 ms). Then, the cuvette was gently flushed with 100 µl SOC medium, and cells were spread onto a nonselective MH agar plate. Plates were incubated for 5 to 6 h at 42 °C under microaerobic conditions to allow for cell recovery. Cells grown on the recovery plate were then harvested in 1 ml of MH broth, centrifuged for 5 min at 5,000 rpm, resuspended in 100 µl of MH broth, and plated onto a MH plate containing 30 µg/ml kanamycin. The plate was incubated at 42 °C under microaerobic condition for 3 to 5 d. Another electroporation reaction with the empty targeting plasmid, pCTarget-cas9 (no spacer sequence), was included as a control for the transformation efficiency of the competent cells and the efficiency of gene editing. The successful replacement of *cmeB* with RE-*cmeB* in the *C. jejuni* NCTC11168 strain was confirmed by PCR using RE-*cmeB-*specific primers (REB-F/R) and *cmeB-*specific primers (11B-F/R) (SI Appendix, Table S2), amplifying 933 bp and 535 bp PCR fragments, respectively (Fig. 3). DNA sequencing was further conducted to verify the replacement.

### Genetic Diversity Analysis of RE-*cmeABC* Carrying *Campylobacter* and Recombination and Evolutionary Analysis of RE-*cmeABC*.

*C. jejuni* and *C. coli* genomic sequences deposited in the NCBI Pathogen Detection Database (https://www.ncbi.nlm.nih.gov/pathogens/) were utilized for the analyses. As of May 10, 2023, 82,371 sequences were available and the genome contigs and metadata of each isolate were downloaded to a local database for processing. The entire region (~6.4 Kb) of *cmeR*-*cmeABC* of each isolate was retrieved from genomes by an in-house Python script. Sequences of low quality (i.e., containing N or degenerate bases, or truncated *cmeABC*) were removed. Then, qualified *cmeR*-*cmeABC* sequences (n = 80,408) were aligned by using MAFFT (65). The alignment was trimmed to only keep the *cmeABC* coding sequences by using Jalview (66) and deduplicated by using SeqKit (67) to reduce the redundancy for phylogenetic analysis. As a result, 8,133 unique *cmeABC* sequences were identified and used to construct an approximately maximum-likelihood phylogenetic tree by using FastTree (68). The *cmeR*-*cmeABC* alignment of 80,408 sequences was further trimmed to only keep the *cmeB* region and construct approximately-maximum-likelihood phylogenetic tree by using Fasttree (68). The phylogenetic trees were visualized in Microreact (69) and correlated to their metadata to analyze the phylogenetic relationship and distribution of RE-*cmeABC* and RE-*cmeB* in *Campylobacter* species. Whole genome sequences of 1510 RE-*cmeB* carrying isolates were analyzed for their STs and antibiotic resistance determinants by using Staramr (70). Minimum spanning tree (MST) of the identified STs was constructed by using PHYLOViZ (71). Recombination analysis of RE-*cmeABC* was performed by using Gubbins (36) and visualized by using Phandango (72). Positive selection was performed by using neutrality tests (Fu and Li’s D*, and Fu and Li’s F*, and MDK test) in DnaSP version 6 (73).

### Disclaimer.

The views expressed in this article are those of the authors and do not necessarily reflect the official policy of the Department of Health and Human Services, the US Food and Drug Administration. The mention of trade names or commercial products in this publication is solely for the purpose of providing specific information and does not imply a recommendation or endorsement by the US Department of Agriculture or the Food and Drug Administration.

## Supplementary Material

Appendix 01 (PDF)

Dataset S01 (XLSX)

Dataset S02 (XLSX)

Dataset S03 (XLSX)

## Data Availability

All study data are included in the article and/or supporting information.
