# Supplementary material for: Mutation-based mechanism and evolution of the potent multidrug efflux pump RE-CmeABC in Campylobacter
Source: Proc Natl Acad Sci U S A. 2024 Nov 27;121(51):e2415823121. doi: 10.1073/pnas.2415823121 (PMC11665921; doi:10.1073/pnas.2415823121)
Supplement: Supplementary file 1 — Appendix 01 (PDF) [file pnas.2415823121.sapp.pdf]

PNAS

Supporting Information for:

Mutation-based mechanism and evolution of the potent multidrug efflux pump RE-CmeABC in *Campylobacter*

Lei Dai, Zuowei Wu, Orhan Sahin, Shaohua Zhao, Edward W. Yu, and Qijing Zhang

Corresponding author: Qijing Zhang
Email: zhang123@iastate.edu

This PDF file includes:

Figures S1 to S5
Tables S1 to S2
Legends for Datasets S1 to S3

Other supporting materials for this manuscript include the following:

Datasets S1 to S3

Lipman-Pearson Protein Alignment

Ktuple: 2; Gap Penalty: 4; Gap Length Penalty: 12

Seq1(1>1040) 11168_CmeB	Seq2(1>1039) DH161_CmeB	Similarity Index	Gap Number	Gap Length	Consensus Length
(1>1040)	(1>1039)	80.9	1	1	1040

```
                     v10       v20       v30       v40       v50       v60       v70       v80       v90       v100      v110      v120
11168_CmeB  MFSKFFIERPVFASVVAIIISLAGAIGLTNLPIEQYPSLTPPTVKVSATYTGADAQTIASTVASPIEDAINGADNMIYMDSTSSSSGTMSLTVYFDIGTDPDQATIDVNNRISAATAKMP
            ||||||||||:||||||||||:|| |||:|||:|||||||||||:||||||||||||||||||:||||||||.|||||||||||: | |.|||||:||||||||:|||||||||||||:|
DH161_CmeB  MFSKFFIERPIFASVVAIIISIAGIIGLANLPVEQYPSLTPPTVQVSATYTGADAQTIASTVATPIEDAINGVDNMIYMDSTSSP-GQMKLTVYFNIGTDPDQAAIDVNNRISAATAKLP
                     ^10       ^20       ^30       ^40       ^50       ^60       ^70       ^80        ^90       ^100      ^110

                     v130      v140      v150      v160      v170      v180      v190      v200      v210      v220      v230      v240
11168_CmeB  DAVKKLGVTVRKTSSTTLAAISMYSSDGSMSAVDVYNYITLNVLDELKRVPGVGDANAIGNRNYSLRIWLKPDLLNKFGITATDVISAVNDQNAQYATGKIGEEPVTQKSPYVYSITMQG
            :|||||||||||:|||.|..:|:||.|:||:.:|:|||::||:||||||:||||||:||||:|||:||||.||||||||:||.|||:|||||||||||||||||||.:||| | ||||||
DH161_CmeB  EAVKKLGVTVRKSSSTILEVVSVYSEDSSMNDIDIYNYVSLNILDELKRIPGVGDASAIGNKNYSMRIWLEPDLLNKFGVTANDVINAVNDQNAQYATGKIGEEPVVNKSPQVISITMQG
            ^120      ^130      ^140      ^150      ^160      ^170      ^180      ^190      ^200      ^210      ^220      ^230

                     v250      v260      v270      v280      v290      v300      v310      v320      v330      v340      v350      v360
11168_CmeB  RLQNPSEFENIILRTNNDGSFLRLKDVADVEIGSQQYSSQGRLNGNDAVPIMINLQSGANALHTAELVQAKMQELSKNFPKGLTYKIPYDTTKFVIESIKEVVKTFVEALILVIIVMYMF
            |||.| ||||||||.|:| ||||:||||.||||::||:| ||||.:.||||:|||||||||::||.|::.||||||||||:||.|:|||||| || .|||||:||||||| ||::|||:|
DH161_CmeB  RLQTPQEFENIILRVNEDKSFLRIKDVAKVEIGAEQYNSTGRLNTSAAVPIIINLQSGANAVNTAKLINEKMQELSKNFPQGLKYQIPYDTTIFVKASIKEVIKTFVEALALVLVVMYLF
            ^240      ^250      ^260      ^270      ^280      ^290      ^300      ^310      ^320      ^330      ^340      ^350

                     v370      v380      v390      v400      v410      v420      v430      v440      v450      v460      v470      v480
11168_CmeB  LKNFRATLIPMIAVPVSLLGTFAGLYVLGFSINLLTLFALILAIGIVVDDAIIVVENIDRILHENEQISVKDAAIQAMQEVSSPVISIVLVLCAVFVPVSFISGFVGEIQRQFALTLAIS
            ||||::|:||||||||||||||| ||||||||||||||||:|||||||||||||||||||||||:.:||||||||:||:|||||||||||||||||:||||||||||||||||||||||||
DH161_CmeB  LKNFKSTIIPMIAVPVSLLGTFAVLYVLGFSINLLTLFALVLAIGIVVDDAIIVVENIDRILHEDSNISVKDAAIKAMNEVSSPVISIVLVLCAVFIPVSFISGFVGEIQRQFALTLAIS
            ^360      ^370      ^380      ^390      ^400      ^410      ^420      ^430      ^440      ^450      ^460      ^470

                     v490      v500      v510      v520      v530      v540      v550      v560      v570      v580      v590      v600
11168_CmeB  VTISGFVALTLTPSLCALFLRRNEGEPFKFVKKFNDFFDWSTSVFSAGVAYILKRTIRFVLIFCIMLGAIFYLYKAVPSSLVPEEDQGLMIGIINLPSASALHRTISEVDHISQEVLKTN
            |:|||||||||||||.|||| |||:.|| |::||||||||||||||:||||||||||||||:||||:| | ||:| |||||||.||||::::||||||.|::||||.||| |:::. : :
DH161_CmeB  VAISGFVALTLTPSLSALFLTRNESKPFYFIQKFNDFFDWSTSVFSSGVAYILKRTIRFVLVFCIMIGFIAYLFKIVPSSLVPSEDQGVIMSIINLPSGSSIHRTIEEVDTINKNATQMK
            ^480      ^490      ^500      ^510      ^520      ^530      ^540      ^550      ^560      ^570      ^580      ^590

                     v610      v620      v630      v640      v650      v660      v670      v680      v690      v700      v710      v720
11168_CmeB  GVKDAMAMIGFDLFTSSLKENAAAMFIGLKDWKDRNVSADEIAMELNKKFAFDRNASSIFIGLPPIPGLSITGGFEMYVQNKSGKSYDEIQKDVNKLVAVANQRKELSRVRTTLDTTFPQ
            .:...:::||||||||||||||||:|: ||||.:|:.|:|:|  :|  ::| |||| | |:.||||||||:|||||||.||||||.||.||:||||::.:|. ||||:.|||||||:|||
DH161_CmeB  EISSSVSLIGFDLFTSSLKENAAAVFFILKDWSQREASSDQIIAQLFGQYAADRNALSYFLNLPPIPGLSLTGGFEMYAQNKSGKDYDAIQQDVNKMLELARTRKELANVRTTLDTSFPQ
            ^600      ^610      ^620      ^630      ^640      ^650      ^660      ^670      ^680      ^690      ^700      ^710

                     v730      v740      v750      v760      v770      v780      v790      v800      v810      v820      v830      v840
11168_CmeB  YKLIIDRDKLKHYNLNMQDVFNTMNATIGTYYVNDFSMLGKNFQVNIRAKGDFRNTQDALKNIFVRSNDGKMIPLDSFLTLQRSSGPDDVKRFNLFPAAQVQGQPAPGYTSGQAIEAIAQ
            |||||||||:|.|||||||||||::|||||||||||:|||||||||||| |||||||||||||::||:|.|||||:||||| ||:|||||||||||||| :||:|||||||||||:|||:
DH161_CmeB  YKLIIDRDKMKYYNLNMQDVFNTISATIGTYYVNDFPMLGKNFQVNIRALGDFRNTQDALKNIYIRSSDNKMIPLNSFLTLVRSAGPDDVKRFNLFPAALIQGDPAPGYTSGQAIDAIAE
            ^720      ^730      ^740      ^750      ^760      ^770      ^780      ^790      ^800      ^810      ^820      ^830

                     v850      v860      v870      v880      v890      v900      v910      v920      v930      v940      v950      v960
11168_CmeB  VAKETLGDDYSIAWSGSAYQEVSSKGTASYAFALGMIFVFLILAAQYERWLIPLAVVTAVPFAVFGSFLLVYLRGFSNDIYFQTGLLLLIGLSAKNAILIVEFAMEERFKKGKGVFEAAV
            |||::|||:|||||||||||||||||..:|||.||||||||||||||||||:||||:||||||||||.||| ||||:|||||||||||||||||||||||:|||||||:||||::||||:
DH161_CmeB  VAKQSLGDEYSIAWSGSAYQEVSSKGAGAYAFVLGMIFVFLILAAQYERWLMPLAVITAVPFAVFGSILLVALRGFDNDIYFQTGLLLLIGLSAKNAILIIEFAMEERLKKGKSIFEAAI
            ^840      ^850      ^860      ^870      ^880      ^890      ^900      ^910      ^920      ^930      ^940      ^950

                     v970      v980      v990      v1000     v1010     v1020     v1030     v1040
11168_CmeB  AAAKLRFRPIIMTSLAFTFGVLPMIFATGAGSASRHSLGTGLIGGMIAASTLAIFFVPLFFYLLENFNEWLDKKRGKVHE
            .|||||||||||||||||||||||||||||||||||||||||||||||||||||||||||||||||||||||||||||||
DH161_CmeB  NAAKLRFRPIIMTSLAFTFGVLPMIFATGAGSASRHSLGTGLIGGMIAASTLAIFFVPLFFYLLENFNEWLDKKRGKVHE
            ^960      ^970      ^980      ^990      ^1000     ^1010     ^1020     ^1030
```

Fig. S1. Lipman-Pearson amino acid alignment of *C. jejuni* NCTC 11168 CmeB and *C. coli* DH 161 RE-CmeB protein sequences. Amino acids in red color represent the differed amino acids between CmeB and RE-CmeB. Amino acids that are uniformly detected (> 99.5 % transfer frequency) in wild-type NCTC11168 and 11168Δ*cmeR* transformants are labeled by black (pointing down) and purple (pointing up) arrows, respectively. Amino acids in red boxes represents the uniformly detected amino acids in both NCTC11168 and 11168Δ*cmeR* transformants. The region under strong positive selection (value<-5, P<0.01, in both Fu and Li's D* and Fu and Li's F* test) is indicated by a green dash box.

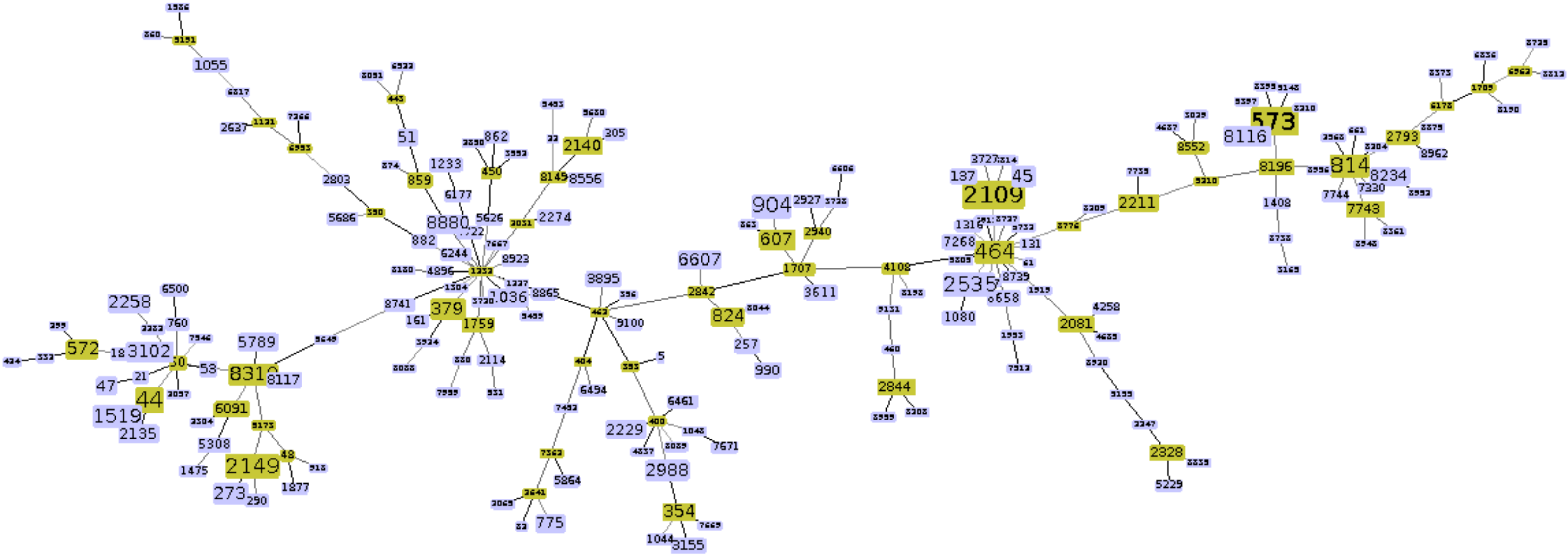

1055
2637
2803
5686
51
859
1233
6177
862
450
2140
305
8556
2274
8880
5626
882
6244
8923
4896
8741
161
379
1759
2114
1036
8865
3895
9100
6494
5864
775
6461
2229
7671
2988
354
1044
3155
6607
2842
824
257
990
904
2927
607
2940
1707
3611
4108
2844
137
3727
45
2109
1316
7268
464
131
2535
8739
1080
2081
4258
2328
5229
2211
8552
8116
573
8196
1408
814
8234
7744
7330
7743
2793
8962
2258
6500
760
572
3102
5789
53
8310
8117
47
21
44
1519
2135
6091
5308
1475
2149
48
273
290
1877

Fig. S2. Minimum spanning tree of the identified 214 STs showing the genetic diversity of RE-*cmeB* positive *C. jejuni* and *C. coli*. The tree was constructed using MLST alleles of RE-*cmeB* harboring isolates. Font size indicates the proportion of the isolates of each ST in the collection. Font colors: yellow depicts double locus variant while light blue indicates single locus variants.

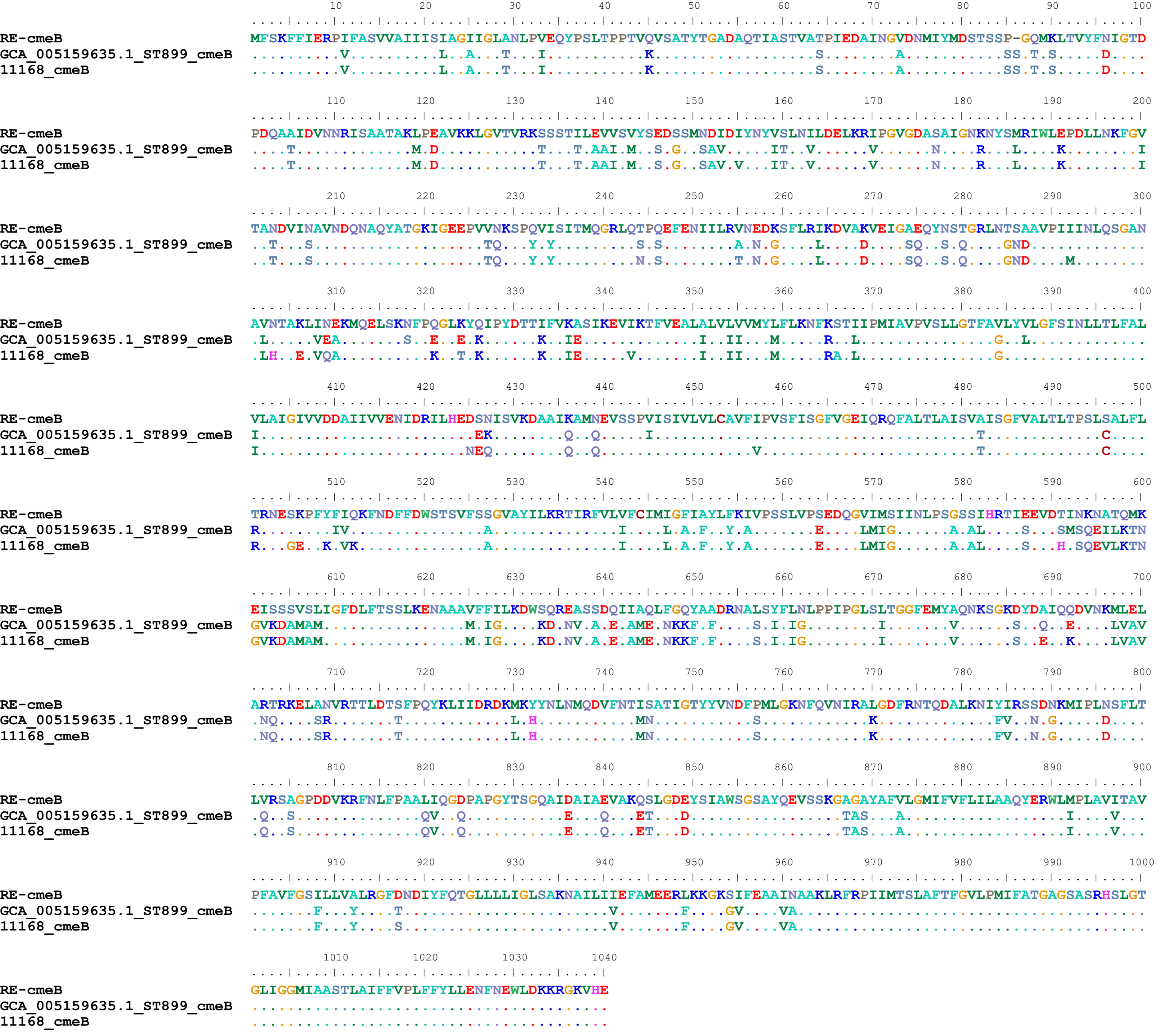
RE-cmeB
GCA_005159635.1_ST899_cmeB
11168_cmeB

Fig. S3. Amino acid alignment of the typical CmeB from strain NCTC11168 (GenBank: NC_002163), CmeB from a ST899 strain (GenBank: GCA 005159635.1), and RE-CmeB (GenBank: KT778507.1).

A

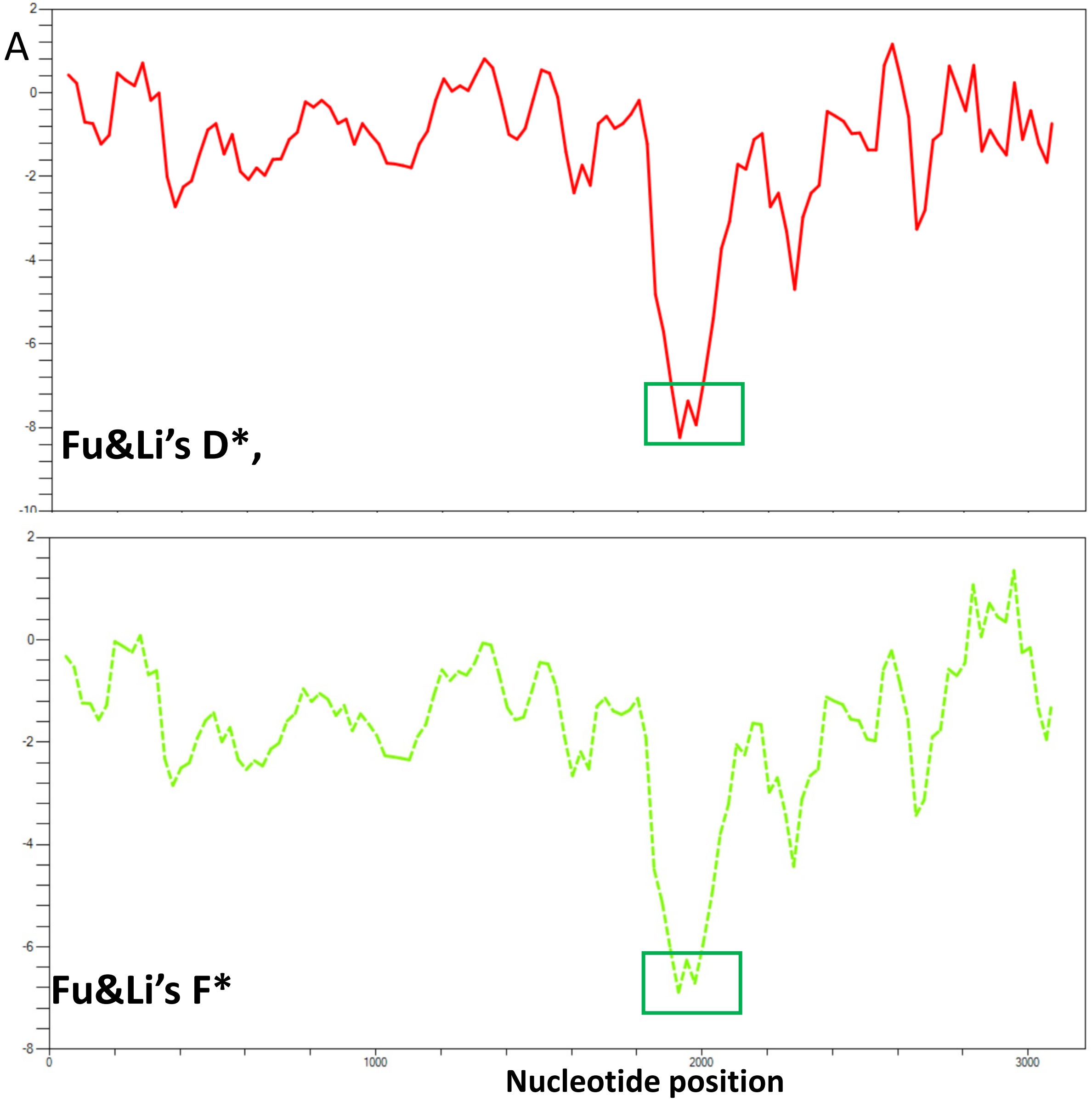

B

Window on RE-*cmeB* CDS	Midpoint	D*	Significance	F*	Significance
1804-1903	1853	-4.8117	**	-4.4803	**
1829-1928	1878	-5.7026	**	-5.1326	**
1854-1953	1903	-7.0358	**	-6.0479	**
1879-1978	1928	-8.2333	**	-6.8853	**
1904-2003	1953	-7.3601	**	-6.2621	**
1929-2031	1978	-7.9327	**	-6.7075	**
1954-2056	2003	-6.8169	**	-5.9583	**
1979-2081	2031	-5.3888	**	-4.9559	**
2004-2106	2056	-3.7158	**	-3.7918	**
2032-2131	2081	-3.0686	*	-3.2137	**
2057-2156	2106	-1.7066		-2.0467	#
2082-2181	2131	-1.8239		-2.2482	#
2107-2206	2156	-1.1150		-1.6288	
2132-2231	2181	-0.9741		-1.6565	
2157-2256	2206	-2.7223	*	-2.9802	**
2182-2281	2231	-2.3967	*	-2.6935	*
2207-2306	2256	-3.3124	**	-3.4104	**
2232-2331	2281	-4.6955	**	-4.4287	**
2257-2356	2306	-2.9695	*	-3.1191	**
2282-2381	2331	-2.3967	*	-2.6603	*
2307-2406	2356	-2.2098	#	-2.5272	*

Fig. S4. Selective sweeps detected by population genetic methods. (A) Nucleotides of RE-cmeB predicted by Fu&Li’s D*, Fu&Li’s F tests (100 bp window, 25 bp stepwise) to be under positive selection (negative values, * 0.01 < p < 0.05;** p < 0.01). The selection values were plotted in the graph against the nucleotide position of the full length of RE-cmeB. The green box indicates the region involved in drug recognition and binding. (B) Enlarged visualization of the statistic windows covering the amino acids in the drug-binding pocket. The strongest signals for positive selection (value<-5, p<0.01 in both Fu and Li's D* and Fu and Li's F* test) were highlighted in green.

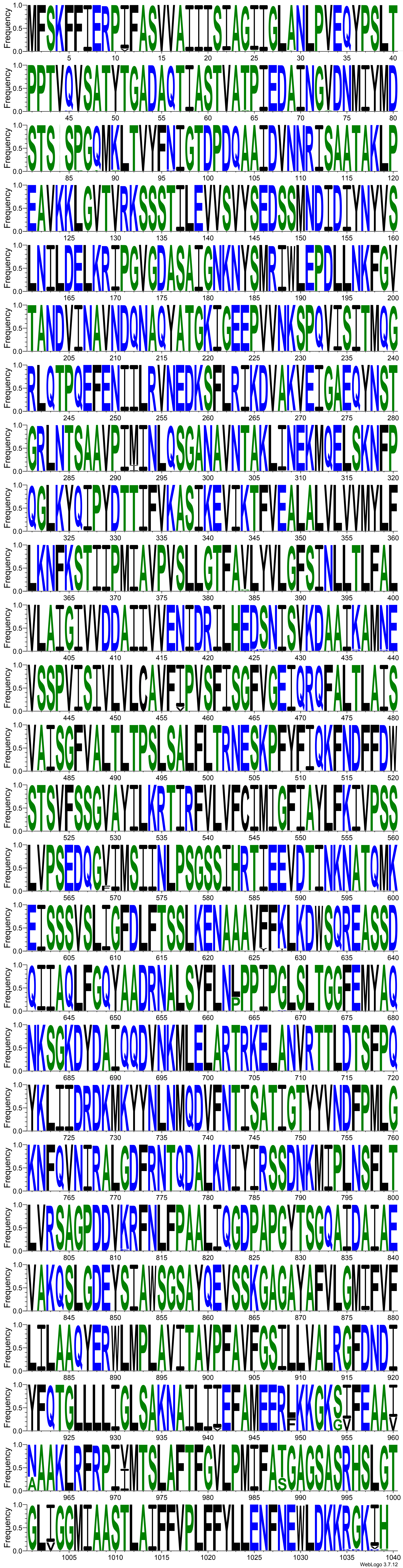
Frequency
WebLogo 3.7.12

Fig. S5. The protein sequence logo of RE-CmeB from 1,510 isolates shows low sequence variation of RE-CmeB. The log was generated by WebLogo 3 based on the protein sequence alignment of RE-CmeB from the RE-*cmeB* clade (1,510 isolates). Y: frequency of an amino acid at each site. X: position of the amino acid in the full-length alignment of RE-CmeB. The color of each amino acid (one letter code) was assigned by Weblogo 3 to indicate its Hydrophobicity (blue-Hydrophilic, green-Neutral, and black-Hydrophobic). A few RE-CmeB sequences had an insertion at position 84, which is indicated by a narrow letter.

Table S1. Antimicrobial MICs (µg/ml) of RE-cmeB positive ST-2109 isolates examined in this study*

Strain No.	MLST type	MIC of different antimicrobials (µg/ml)								
		AZI	CLI	CIP	ERY	FFN	GEN	NAL	TEL	TET
1	ST-2109	> 64	>16	32	> 64	4	> 32	> 64	> 8	> 64
2	ST-2109	> 64	8	32	> 64	4	> 32	> 64	> 8	> 64
3	ST-2109	> 64	> 16	32	> 64	8	> 32	> 64	> 8	> 64
4	ST-2109	> 64	8	32	> 64	4	> 32	> 64	> 8	> 64
5	ST-2109	> 64	8	32	> 64	4	> 32	> 64	> 8	> 64
6	ST-2109	> 64	8	32	> 64	4	> 32	> 64	> 8	> 64
7	ST-2109	> 64	8	64	> 64	4	> 32	> 64	> 8	> 64
8	ST-2109	> 64	4	32	> 64	4	> 32	> 64	> 8	> 64
9	ST-2109	> 64	4	32	> 64	4	0.5	> 64	> 8	> 64
10	ST-2109	> 64	8	32	> 64	4	> 32	> 64	> 8	> 64

*AZI, Azithromycin; CLI, Clindamycin; CIP, Ciprofloxacin; ERY, Erythromycin; FFN, Florfenicol; GEN, Gentamicin; NAL, Nalidixic acid; TEL, Telithromycin; TET, Tetracycline

Table S2. . Primers used in this study

Primers	Sequences
REcmeABC-F	TTCAATCAACCAGAAGCTGT
REcmeABC-R	GTTTAAACAAAGGGCGGAAT
pRRK-lF	TTTCAGTAACCAAACTATACATATTGGTTTTAGAATGCAAGGAAC
pRRK-lR	AGTCCATTTTTTTTAGCACACTCATTCTAGAAAGGAGGTGATCC
cjCRISPR-F	ATGAGTGTGCTAAAAAAAATG
cjCRISPR-R	AATATGTATAGTTTGGTTACTGAAAAG
pCTarget-cas9-F	TCATTTTTTAAAATCTTCTCTTTGTC
pCTarget-cas9-R	TTAAAAGCGGTTTTAGGG
cmeB-sp-F	[*]**taaagaatttccg**gttttagtcccttttaaatTTCTTTATGGTAAAATAGATATTTAC
cmeB-sp-R	[*]**agaacatatacataaca**attttaccataaagaaATTTAAAAAGGGACTAAAACATTTAAG
cmeA-F	TAGTGTTGATTCGGCTTACG
cmeA-R	tttagaaaacatTATTGTGCTCCAATTTCTTTAAC
REcmeB-F	gcacaataATGTTTTCTAAATTTTTTATAGAAAGACC
REcmeB-R	ctaattgaaattattTTATTCATGAACCTTACCTCTTTTTTTATC
cmeC-F	atgaataaAATAATTTCAATTAGTGCTATAGCAAG
cmeC-R	AATATCTGGACGTTGAAGCA
pUC18-lF	tgcttcaacgtccagatattTCTAGAGGATCCCCGGGTAC
pUC18-lR	cgtaagccgaatcaacactaGTCGACCTGCAGGCATGC
cmeAP-F	AGTTGTTATCAGGGCTACAAA
cmeAP-R	CCATTTTGAGTATTGCGGAC
pUC18-lF1	gtccgcaatactcaaaatggTCTAGAGGATCCCCGGGTAC
pUC18-lR1	ttgtagccctgataacaactGTCGACCTGCAGGCATGC
cmeAPG-F	AAATTTTTGTgATAAAAATTACAATTTTTAATTTAATTTTTC
cmeAPG-R	TTGGCTAATTATATCTTAATTTTG
REB-F	AAGTAGAAATTGGGGCAGAG
REB-R	AGCCAGAAGGAAGGTTTATG
11B-F	TGGAGTAACAGTTAGAAAAACTTCC
11B-R	TGTATGCAATGCGTTTGCCC

[*] lower case letters in bold represent spacer sequences from the *cmeB* gene; lower case letter underlined represent sequences of an additional NCTC11168 crRNA repeat unit to be added; upper case letters represent sequences of the existing crRNA repeat unit on the pCTarget-*cas9* or pCTarget plasmid.

Dataset S1 (separate file). Amino acid point mutations that are uniformly detected in the antibiotic-resistant transformants.

Dataset S2 (separate file). Transfer frequencies of various SNPs in RE-*cmeB* to the antibiotic-resistant transformants of *C. jejuni* NCTC 11168 and 11168Δ*cmeR*.

Dataset S3 (separate file). Metadata of 1,510 *Campylobacter* isolates carrying RE-*cmeABC* from the NCBI database
